# Benchmarking Protein–Ligand Cofolding Models: Correct Ligand Placement Is Decoupled From Accurate Protein Conformation

**DOI:** 10.1021/acs.jcim.6c02169

**Published:** 2026-10-20

**Authors:** Leon Obendorf, Niklas Piet Doering, Petra Knaus, Gerhard Wolber

**Affiliations:** † Department of Biology, Chemistry and Pharmacy, Institute of Biochemistry, Signal Transduction Group, 9166 Freie Universität Berlin, Thielallee 64, Berlin 14195, Germany; ‡ Department of Biology, Chemistry and Pharmacy, Institute of Pharmacy, Molecular Design Group, Freie Universität Berlin, Königin-Luise-Str. 2 + 4, Berlin 14195, Germany

## Abstract

AI-driven cofolding models have become central tools for predicting protein–ligand complexes, yet it remains unclear whether the resulting structures recover the correct protein conformation or merely place the ligand plausibly within an otherwise mispredicted fold. Here, we benchmark four state-of-the-art cofolding models, AlphaFold3, RoseTTAFold3, Boltz-2, and Chai-1, and show that correct ligand placement is systematically decoupled from accurate prediction of the surrounding protein conformation. As test systems, we use kinases and Class A G protein-coupled receptors (GPCRs), families whose pharmacology depends on well-defined conformational states. To enable a prospective assessment, we evaluate complexes released after the training cutoffs of all tested models, together with one earlier nucleotide-bound structure as a control. We find that state-defining structural features, including kinase activation-loop, DFG/DYG geometries, and GPCR intracellular conformations such as ICL3, are frequently mispredicted even when ligand root-mean-square deviation (RMSD) is low. Cofolding adaptively remodels the orthosteric pocket around the bound ligand, but this local adaptation rarely propagates to the correct global conformation. Incorporating state-annotated templates and filtered multiple sequence alignments (MSAs) improves conformational recovery in some cases but has little effect in others, and model confidence metrics correlate with small-molecule placement but cannot report whether the local pocket geometry was recovered. Furthermore, while orthosteric ligand placement is generally reliable, the single allosteric complex in our set is misplaced by all models, even under explicit guidance. These results identify conformational decoupling as a fundamental limitation of current cofolding approaches across the systems evaluated, with direct implications for state-selective and structure-based drug design.

## Graphical abstract

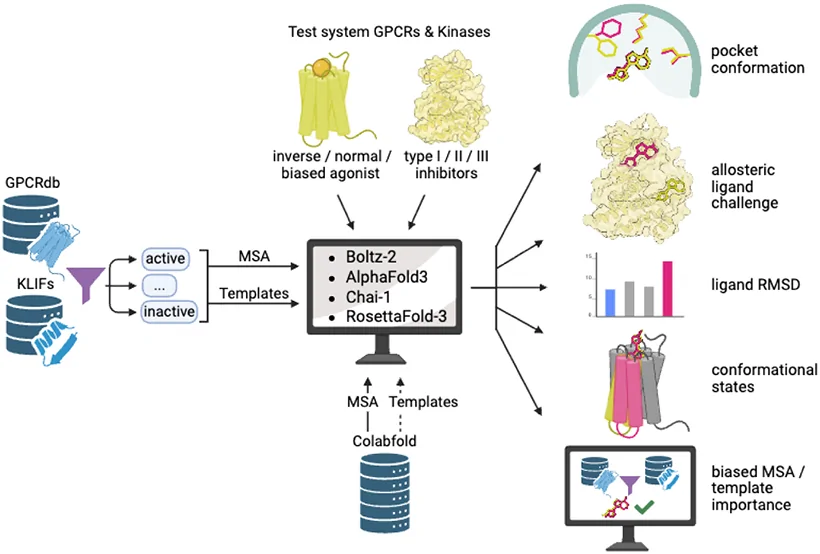

## Introduction

Proteins act as dynamic macromolecules that reside in an ever changing conformational ensemble.^1^ This ensemble often determines physiological outcomes and can be modified by ligand-binding events. Kinases^2^ and G protein-coupled receptors (GPCRs)^3^ are two well-known protein families where these shifts in conformational ensembles guide activity and downstream signaling. Although kinases share a conserved bilobal fold, they differ in how active and inactive states are achieved, often through small shifts in the activation loop, the DFG motif, the αC-helix or nearby regulatory elements that affect substrate binding.^4^ In GPCRs, signaling arises from rearrangements of conserved motifs (e.g., DRY, NPxxY, PIF) and a reshaping of the intracellular binding pocket to open for transducer binding.^3^ When aiming to work with structural data of kinases or GPCRs in *in silico* investigations, it is crucial to model the correct state for the bound ligands.

Although deep learning models have transformed structural biology through *de novo* protein structure generation (protein folding), widely used predictors such as AlphaFold2 (AF2) often default to the most common conformations present in their training sets.^5^ For systems that adapt significantly different states, this can lead to problems such as prediction in an unwanted state or even prediction in mixed states that represent a nonphysiological average of all states within the training set.^5^

Methodological advances aim to address state ambiguity in deep-learning structure prediction by biasing protein prediction models toward user-specified functional conformations. One widely used strategy is template filtering, in which inputs are restricted to state-annotated experimental structures from curated resources such as GPCRdb (https://gpcrdb.org/)^6^ and KLIFS (https://klifs.net/),^7^ enabling predictions to be steered toward desired conformations.^8^ A complementary approach modulates the evolutionary signal contained in multiple sequence alignments (MSAs): reducing MSA depth or clustering sequences can weaken dominant coevolutionary patterns and expose alternative structural states, including inactive and DFG-out kinase conformations^9,10^ and alternative states of GPCRs.^11^ Importantly, these two strategies are not independent. Heo and Feig demonstrated that state-annotated templates alone are insufficient when deep MSAs are present, whereas no or shallow MSA sampling allows templates to reliably enforce active and inactive GPCR states with near-experimental accuracy.^5^ This combination was also shown for kinases to outperform template-only input, particularly in flexible or poorly conserved regions.^8^ Together, these studies show that state-aware prediction is achievable, but remains highly sensitive to careful manual template selection combined with MSA control rather than emerging robustly from the models themselves.

In recent developments, cofolding of small molecules with proteins was made possible by AlphaFold3 (AF3)^12^ and soon Boltz-2,^13,14^ Chai-1 ^15^ and recently RoseTTAFold3 (RF3)^16^ followed. Trained on diverse biomolecular interactions, they are supposed to be able to geometrically model induced fit and extend the rigid-body assumptions of conventional docking. In benchmarks such as ″PoseBusters″,^17^ these models achieved success rates that surpass widely used docking tools like AutoDock Vina^18^ and GOLD.^19,20^ However, these gains primarily reflect improved geometric placement rather than explicit learning of physical interaction energies. As shown by Masters et al.,^21^ systematic perturbation of binding sites often failed to alter predicted ligand poses, with models placing ligands in canonical positions even after mutations abolished the pocket. Very recent benchmarks performed in parallel to the present study further report that cofolding models place orthosteric ligands reliably but fail systematically on allosteric sites,^22^ that kinase conformational states are recovered in only a fraction of predictions despite strong ligand-placement scores,^23^ and that AlphaFold3 enzyme conformations can reflect PDB memorization more than ligand-induced switching.^24^ These studies quantify how default settings fail, but not whether that failure can be corrected. This motivates testing whether state-biasing strategies developed for classical protein folding, such as MSA manipulation and template selection, can enforce state-specific geometry in cofolding.

We systematically assessed the practical capabilities and limitations of current protein–ligand cofolding approaches in state-dependent systems using four state-of-the-art models: AlphaFold3,^12^ Boltz-2,^13,14^ Chai-1,^15^ and RF3.^16^ We curated a benchmark of kinases and Class A GPCRs, spanning distinct functional states that were released after the training cutoffs of the evaluated models, enabling prospective rather than retrospective evaluation, together with one earlier nucleotide-bound complex included as a control. Controlled template and MSA biasing strategies, informed by state annotations from KLIFS^7^ and GPCRdb,^6^ were applied throughout. For kinases, we selected structures that were recently solved and comprise all three inhibitor types: LRRK2 bound by the type II inhibitor RN277 (PDB: 9DMI),^25^ the PI3K^H1047R^ mutant bound by the allosteric inhibitor zovegalisib within a cryptic pocket (PDB: 8TSD),^26^ and the active-like state of activin-like kinase ALK2 bound by the type I inhibitor RK783 (PDB: 9L04),^27^ supplemented by an AMPPNP-bound ALK2 structure to probe nucleotide recognition beyond inhibitor-induced states (PDB: 6UNQ).^28^ The GPCR subset comprised three Class A receptors spanning inactive and active signaling states: the antagonist-bound 5-HT_2A_ receptor, the agonist-bound adenosine A_2A_ receptor, and the δ-opioid receptor bound to a G protein-biased agonist, systems that require simultaneous recovery of orthosteric ligand placement and the intracellular geometries associated with receptor activation, providing a stringent test of whether ligand guidance alone is sufficient to enforce global conformational states.

## Results

### Ligand Placement is Robust across Cofolding Models but Decoupled from State Accuracy and Pocket Geometry

We first evaluated ligand placement and binding-site geometry in complexes predicted by the cofolding frameworks. Across the evaluated kinase systems, low ligand RMSD did not consistently coincide with correct recovery of binding-site geometry. The first example showing this pattern is LRRK2 bound to the type II inhibitor RN277. Here, across predictions that reproduced the global kinase fold, ligands were positioned consistently (Figure 1 A) and ligand RMSDs were collectively below 1.3 Å. Regarding binding-site geometry, the LRRK2-RN277 reference PDB structure features an upward-oriented conformation of the DYG-motif tyrosine, a geometry not observed in PDB entries released before the 2025 cryo-EM structure. This novel Y2018 orientation (Figure 1 B) was recovered only in a subset of predictions, namely AlphaFold3 with biased inputs. Chai-1 also roughly positioned the side chain of the tyrosine in the correct direction in all conditions where no MSA was applied, but the DYG-motif flipped its backbone orientation in these Chai-1 conditions. Boltz-2 and RoseTTAFold3 instead consistently reproduced the canonical inactive tyrosine arrangement. By simple RMSD calculation, Chai-1 using the no-msa custom-templates condition yielded the lowest ligand RMSD (0.55 Å), although showing incorrect conformations of the DYG motif and a nonphysical chair deformation of a phenyl moiety in RN277 (Figure 1 C), with the latter being present in all Chai-1 predictions.

**Figure 1:**
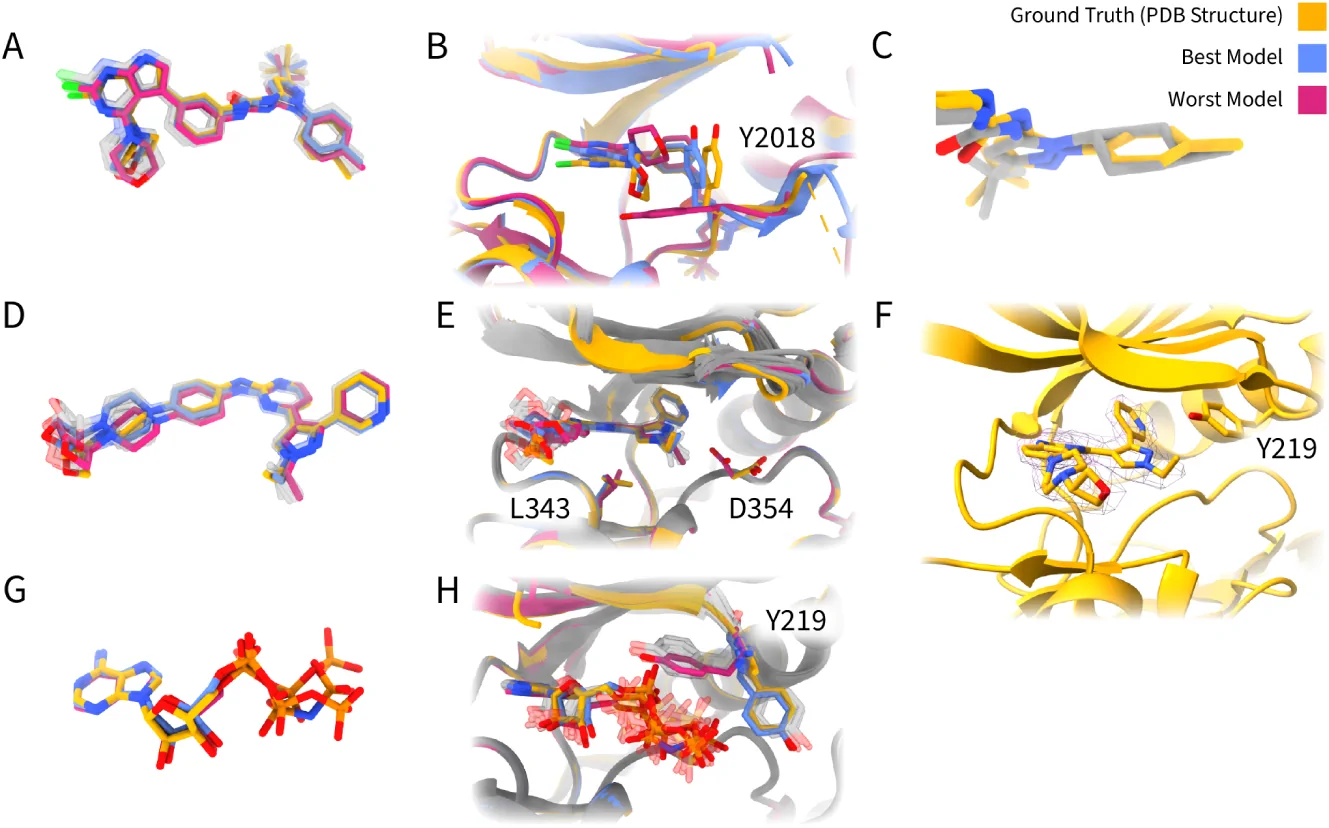
Cofolding predictions of kinases with small molecule inhibitors compared to ground truth structures: **A-C** LRRK2 with the type II inhibitor RN277, **D-F** ALK2 with the type I inhibitor RK783, **G-H** ALK2 with ATP bound. Representative model per condition was chosen based on the highest mean protein pLDDT. As representative conditions one successful and one failed condition was chosen manually by taking into account RMSD, interaction analysis, and pocket-geometry inspection. **A** Superimposition of all predicted RN277 ligand conformations (transparent) with the cryo-EM reference structure (PDB: 9DMI; yellow), highlighting the structurally closest (blue) and farthest (magenta) structure. **B** Close-up view of the DYG-motif with Y2018 shown in stick representation in the predicted structures compared to the reference. **C** Comparison of ground truth (yellow) with Chai-1 predicted ring conformation of RN277. **D** Superimposition of predicted RK783 ligand conformations in ALK2 with the crystal structure reference (PDB: 9L04; yellow), showing differences in to solvent-exposed distal portion of the ligand (lower left) and orientation of an ethyl group. **E** Close-up view of the RK783 binding site with deviations in local pocket geometry in the worst prediction where D354 is rotated toward the ligand, and the 1-ethyl substituent of a pyrazole is facing the lower kinase lobe. **F** Orientation of the experimentally determined 1-ethyl substituent of RK783 relative to the p-loop and Y219 in the ground truth PDB: 9L04. **G** Superimposition of the AMPPNP-bound reference structure (PDB: 6UNQ) and predicted ATP conformations of representative successful/failed conditions. **H** Close-up view of the nucleotide binding pocket showing the conformation of the P-loop residue Y219 relative to the bound nucleotide. The outward-facing Y219 observed in the reference structure is reproduced in a subset of predictions.

Similar for ALK2 bound to the type I inhibitor RK783, models that successfully predicted the kinase fold also generated ligand poses with RMSDs below 1.0 Å (Figure 1 D). Structural deviations in the ligand were localized primarily to solvent-exposed ligand regions and adjacent pocket residues (Figure 1 E). Furthermore, the 1-ethyl substituent of RK783 pointing toward Y219 was recovered in a subset of predictions. Among the successful predictions, AlphaFold3 with biased MSAs and biased templates shows the lowest ligand RMSD (0.70 Å). The Boltz-2 condition with biased MSA + no templates reproduced the ethyl orientation but showed a 180° flip of the pyridine ring in RK783. Chai-1 under the no MSA + no templates condition resulted in incorrect ethyl orientation and rotation of D354 toward the ligand, disrupting a salt bridge observed in the experimental structure between D354–R375 (Figure 1 E). The variation in ethyl-group orientation in the predictions contrasts with the experimental density map, indicating a defined substituent position (Figure 1 F).

To extend the analysis beyond inhibitors, ATP binding to ALK2 was evaluated using the AMPPNP-bound structure (PDB: 6UNQ) as a reference. Based on its release date of 2021–07–07, this structure lies within the training window of AlphaFold3, Boltz-2, and RF3. Ligand RMSDs ranged from 0.61 to 1.62 Å across successful predictions (Figure 1 G), with protein backbone RMSDs below 0.60 Å. A defining feature of the active site is the outward rotation of the P-loop residue Y219, which was reproduced only in a subset of predictions (Figure 1 H), from which none was by Chai-1. Boltz-2 achieved the lowest ligand RMSD (0.61 Å) using standard MSA generation without templates.

Across all three systems, low ligand RMSDs were also observed in models that differed in residue orientation and local interaction networks, indicating that accurate ligand placement can occur without recovery of the experimental binding-site geometry.

### Cofolding Recovers the Correct Protein Fold of Pi3kα but Never Places Zovegalisib in the Allosteric Binding Pocket

To evaluate whether cofolding can recover non-orthosteric binding modes, we analyzed the PI3Kα–H1047R mutant in complex with zovegalisib (RLY–2608), a first-in-class, mutant-selective allosteric inhibitor.^26^ In the experimentally determined structure (PDB 8TSD),^26^ zovegalisib occupies a cryptic allosteric pocket distinct from the canonical ATP-binding site. However, across all models and parameter settings tested, none of the predicted structures placed the ligand in this allosteric pocket; instead, every model positioned it within the ATP binding site (Figure 2), at around 16 Å deviation from the ground truth (Figure 4, bottom panel).

**Figure 2:**
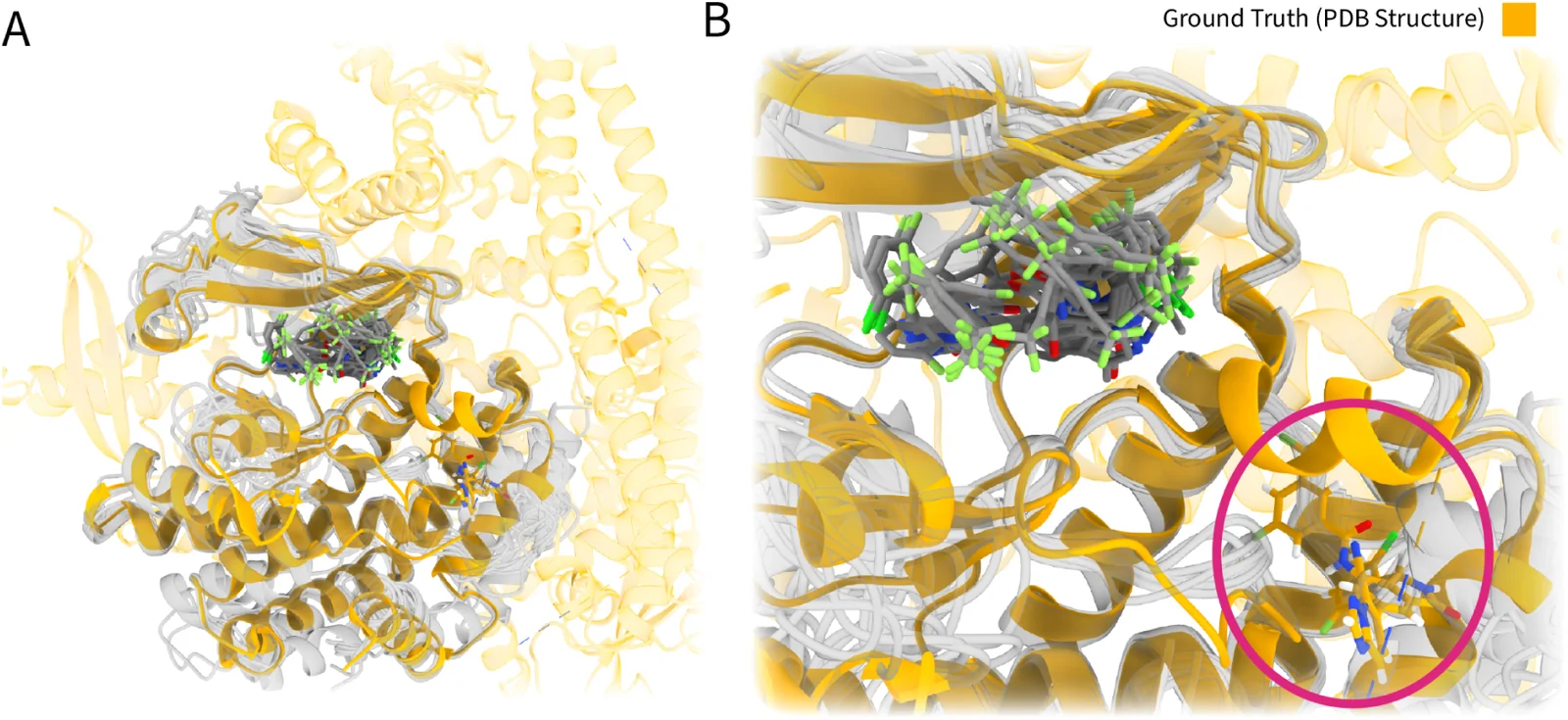
PI3Kα-H1047R mutant in complex with zovegalisib (RLY-2608). Yellow indicates the ground-truth PDB structure, while gray shows all cofolding model predictions. **A** Overview of the general PI3Kα fold. **B** Ligand placement of zovegalisib (RLY-2608). All cofolding models position the ligand in the canonical ATP-binding site, whereas the true ligand occupies a nearby allosteric pocket (red circle).

Despite the incorrect ligand placement, the overall kinase fold was reproduced accurately, and backbone RMSDs were generally 1.9–3.9 Å for the conditions that fold into a typical kinase-fold. Efforts to guide sampling, including blocking the ATP site sterically, introducing known protein interaction partners associated with the allosteric conformation, and using Boltz-2 binding contact flags to bias the ligand toward the cryptic pocket, did not lead to recovery of the allosteric site. All models continued to place the ligand in the canonical ATP pocket. This example may indicate a limitation of current cofolding approaches for predicting ligands that occupy noncanonical sites, even when the overall fold is accurately modeled. This finding is consistent with an independent benchmarking study conducted in parallel with our work, which likewise reported an architecture-independent failure of current cofolding models on allosteric complexes.^22^

### GPCR Ligand Placement Is Decoupled from Intracellular State Recovery

To assess the ability of cofolding models in capturing both ligand binding and receptor conformational changes, we examined three Class A GPCRs representing distinct active and inactive states. GPCRs undergo substantial rearrangements, particularly in intracellular regions such as ICL3, which play key roles in G protein coupling. We selected three representative test cases, the inactive-state 5-HT_2A_ receptor bound to pimavanserin (PDB: 8ZMG)^29^ (Figure 3 A,D), the active-state adenosine A_2A_ receptor bound to CGS21680 (PDB: 8UGW)^30^ (Figure 3 B,E), and the active-state δ-opioid receptor (DOR) bound to the G protein-biased agonist ADL5859 (PDB: 8Y45)^31^ (Figure 3 C,F). Across these examples, a consistent pattern emerged: while ligand placement and pocket adaptation within the orthosteric binding site could often be predicted accurately, the intracellular regions, particularly ICL3, were frequently misfolded, revealing a decoupling between ligand binding and intracellular state recovery.

**Figure 3:**
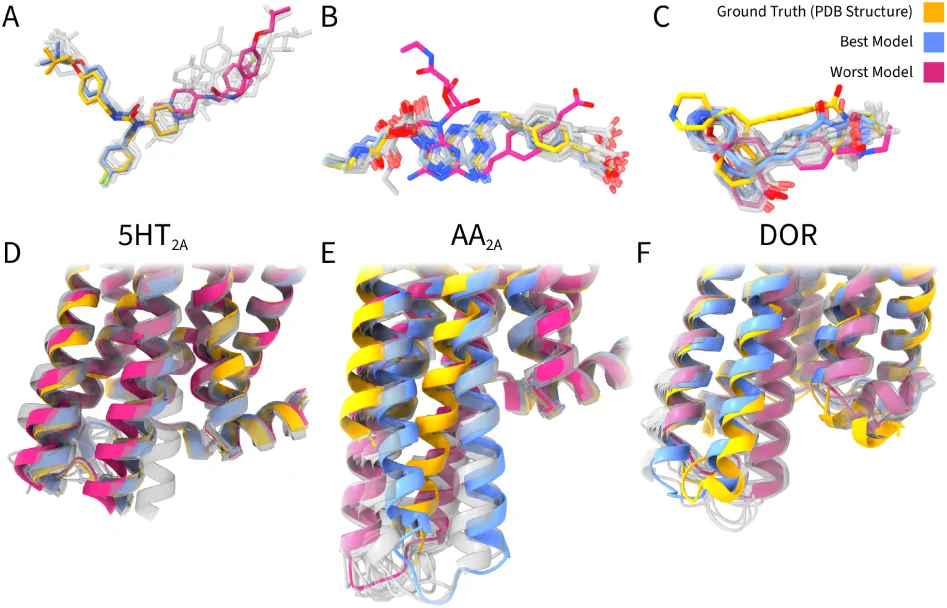
Placement of ligands and ICL3s in the cofolded GPCR structures, showing the highest mean-protein-pLDDT structures. Yellow indicates the reference PDB (ground truth), blue shows the best-performing model based on ligand RMSD, red shows the worst-performing model, and all other predictions are represented in gray. **A** Conformations of the antagonist pimavanserin in the 5-HT_2A_ receptor. **B** Conformations of the agonist CGS21680 in the adenosine A_2A_ receptor. **C** Conformations of the G protein-biased agonist ADL5859 in the DOR. **D** ICL3 folds of the inactive-state 5-HT_2A_ receptor models. **E** ICL3 folds of the active-state A_2A_ receptor models. **F** ICL3 folds of the active-state DOR models.

Across the three GPCRs, ligand placement was generally accurate, with all models not only correctly positioning the ligand within the orthosteric binding site but also, in most cases, reproducing the correct ligand conformation (Figure 3 A-C). For the adenosine A_2A_ receptor, nearly all models produced reasonable ligand placements, with the exception of Chai-1 when using shallow MSAs, which led to less accurate positioning. A notable exception among the predictions were all Chai-1 outputs in the inactive-state 5-HT_2A_ receptor bound to pimavanserin, where only the inclusion of structural templates allowed recovery of the correct ligand pose and conformation (Figure 3 A). In the DOR bound to the G protein-biased agonist ADL5859, all models showed some deviations in ligand positioning, yet the general placement and overall ligand geometry within the binding pocket remained correct (Figure 3 C). These observations indicate that, despite occasional outliers, ligand prediction is often robust across both active and inactive GPCR states.

In contrast to ligand placement, the accuracy of the protein fold varied considerably across receptors. Residues lining the binding pocket were generally well modeled, whereas larger deviations emerged in the intracellular regions, most notably ICL3 and the adjoining cytoplasmic ends of TM5 and TM6, where active- and inactive-state conformations differ most (Figure 3 D-F). The inactive-state 5-HT_2A_ receptor was reproduced accurately across models (Figure 3 D, Table 1), whereas both active-state receptors, the adenosine A_2A_ receptor and the DOR, were modeled considerably less accurately (Figure 3 E,F, Table 1). This was most pronounced in ICL3, where nearly all models failed to recover the active-state conformation and instead adopted an inactive-like fold or a ″hybrid″ configuration (Figure 3 E,F). To quantify this, we related the local backbone RMSD of the intracellular segment spanning the cytoplasmic ends of TM5 and TM6 (X^5.50^-Y^6.50^) to the global receptor RMSD. For both active-state receptors this ratio ranged from 1.5 to 2.1, excluding the generally misfolded AF3 and RF3 predictions without MSA and templates (Table 1), indicating that ICL3 is modeled roughly twice as poorly as the receptor as a whole, while ratios of 0.6 to 1.0 (Table 1) for the inactive-state receptor indicate that the same segment is recovered at least as accurately as the remaining fold. The only notable exception to active-state ICL3 misfolding was the DOR model generated with AlphaFold3 using custom MSAs and templates, which closely approached the correct active-state intracellular geometry (RMSD ratio 0.83). Together, these observations reveal a divergence between robust ligand placement and accurate prediction of the protein fold itself, particularly for active-state GPCRs.

**Table 1:** Backbone RMSD (Å) of the Best Sample per Condition (Lowest Total RMSD)^a^

MSA/Tpl	5-HT_2A_ (inactive)			A_2A_ (active)			DOR (active, biased)		
	Total	ICL3	Ratio	Total	ICL3	Ratio	Total	ICL3	Ratio
AlphaFold3									
cust/cust	1.45	0.93	0.64	2.07	3.89	1.88	2.63	2.19	0.83
cust/–	1.42	0.95	0.67	2.03	3.85	1.89	3.44	6.51	1.89
base	1.38	1.03	0.74	1.27	2.44	1.93	3.45	6.47	1.88
–/cust	1.40	1.07	0.76	1.47	2.99	2.04	2.52	4.35	1.73
–/–	7.00	3.73	0.53	6.25	3.56	0.57	9.15	5.71	0.62
Boltz-2									
srv/tpl	1.01	0.59	0.59	1.64	3.46	2.11	3.51	6.47	1.84
base	1.03	0.74	0.72	2.01	4.20	2.09	3.40	6.36	1.87
bias/tpl	1.06	0.73	0.69	1.34	2.07	1.55	3.28	6.00	1.83
bias/–	1.00	0.61	0.61	1.34	2.66	1.98	3.41	6.35	1.86
–/tpl	1.14	1.10	0.97	1.39	2.10	1.51	3.21	5.70	1.78
–/–	1.09	0.77	0.71	1.53	2.52	1.65	3.36	5.96	1.78
Chai-1									
base	1.11	0.99	0.89	2.00	3.56	1.78	3.32	5.89	1.77
srv/cust	1.24	1.11	0.89	1.96	3.42	1.75	3.17	5.64	1.78
srv/–	1.02	0.82	0.80	2.05	3.76	1.83	3.55	6.66	1.88
cust/cust	1.22	1.12	0.92	1.94	3.32	1.71	3.31	5.95	1.80
cust/–	1.57	1.00	0.64	1.89	2.84	1.50	3.36	6.14	1.83
–/cust	1.11	0.94	0.84	1.26	2.42	1.93	3.37	6.13	1.82
–/–	1.51	0.97	0.65	1.72	3.30	1.91	3.15	5.88	1.87
RoseTTAFold3									
cust/–	1.59	0.99	0.62	1.84	2.84	1.54	3.32	6.58	1.98
base	1.38	0.92	0.66	1.92	3.20	1.67	3.41	6.24	1.83
–/–	12.50	7.16	0.57	9.54	7.75	0.81	14.33	6.47	0.45

a ICL3 = BW 5.50–6.50; Ratio = ICL3/total. Base = recommended base settings; MSA: srv = server, cust = custom, norm = normal, bias = biased; Tpl: cust = custom, srv = server, tpl = template, – = none.

Overall, these results underscore a decoupling between ligand placement and intracellular state recovery. While current cofolding models can often position GPCR ligands correctly within the orthosteric site, they struggle to reproduce the precise intracellular conformations associated with a specific activation state. This has important implications for GPCR modeling, suggesting that cofolding may be useful for guiding ligand-based decisions, as ligand placement can often be achieved even when intracellular loops are mispredicted. However, it does not allow one to reliably infer the functional outcome of a ligand, since accurate modeling of the intracellular signaling interfaces is still largely beyond reach without additional structural guidance.

### Template and MSA Biasing Affect Prediction Quality but Do Not Reliably Enforce Correct Conformations

The importance of either MSA or templates becomes clear across systems as omitting MSAs and templates in AlphaFold3 and RoseTTAFold3 results in globally misfolded structures with backbone RMSD values exceeding 17 Å and misplaced ligands (Figures 4 and 5). Under the same conditions, Chai-1 and Boltz-2 continued to generate overall correct protein folds without the use of MSAs or templates, but frequently exhibited substantial errors in ligand conformation or pocket geometry. In two cases, these issues produced the poorest-quality structures among all non-globally misfolded predictions.

**Figure 4:**
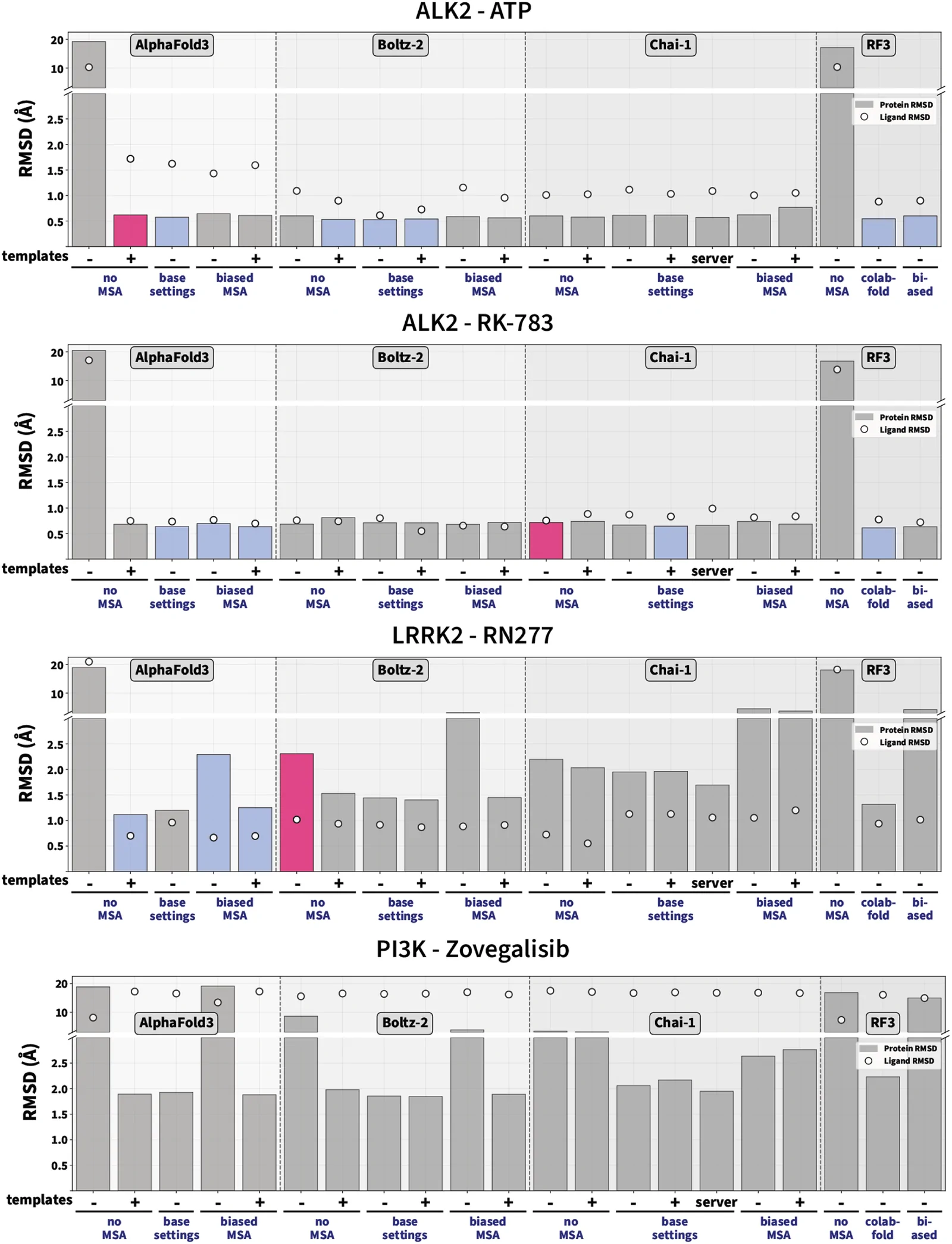
Protein backbone RMSD in Å (bars) and ligand RMSD in Å (dots) for cofolding predictions of kinase systems generated with AlphaFold3, Boltz-2, Chai-1, and RoseTTAFold3 across different MSA and template settings. For each condition, the structure with the highest mean protein pLDDT among the diffusion samples is plotted. Blue bars: close to ground truth predictions chosen by manual comparison of local pocket and ligand geometry in comparison to the ground truth PDB, interaction analysis and low ligand RMSD. In magenta, the structurally most divergent model is shown, considering both ligand RMSD and local environment.

**Figure 5:**
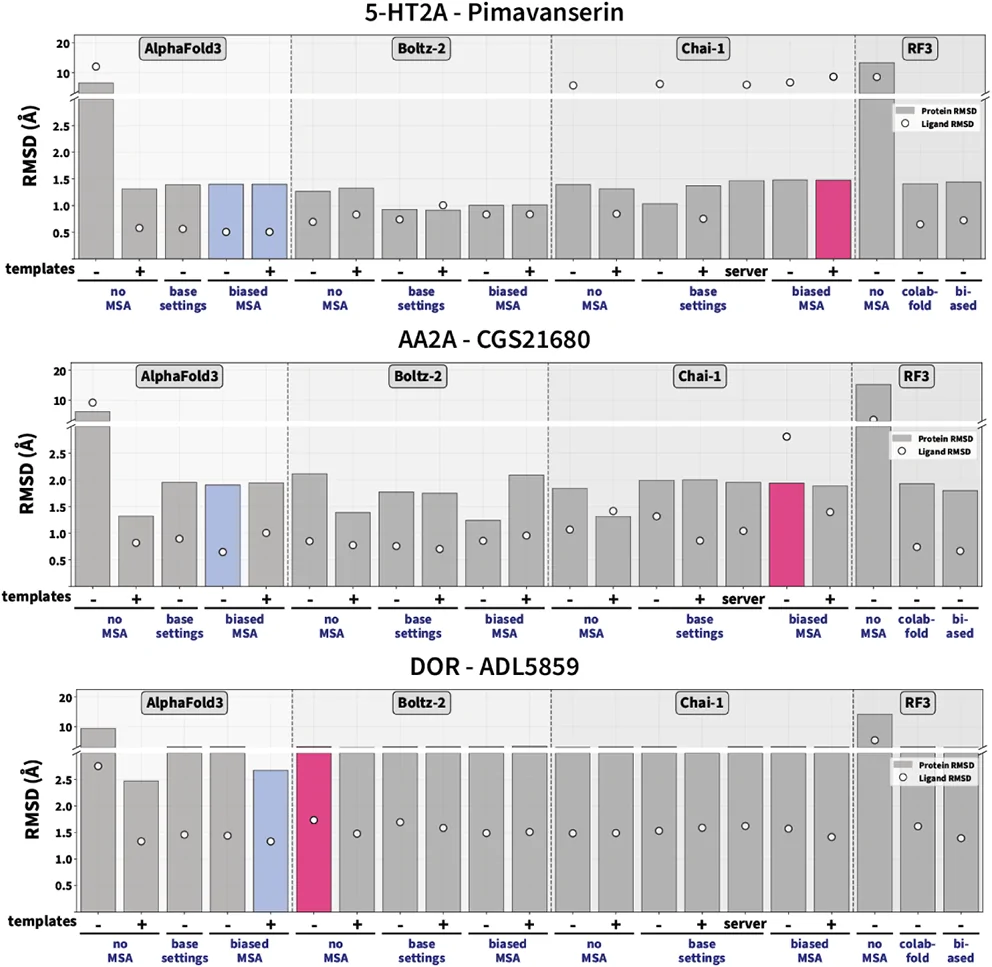
Protein backbone RMSD in Å (bars) and ligand RMSD in Å (dots) for cofolding predictions of GPCR systems generated with AlphaFold3, Boltz-2, Chai-1, and RoseTTAFold3 across different MSA and template settings. For each condition, the structure with the highest mean protein pLDDT among the diffusion samples is plotted. Blue bars: close to ground truth predictions chosen by manual comparison of local pocket and ligand geometry in comparison to the ground truth PDB, interaction analysis and low ligand RMSD. In magenta, the structurally most divergent model is shown, considering both ligand RMSD and local environment.

Ligand RMSD was low across most conditions whenever the global fold was reproduced (Figures 4 and 5), yet it does not discriminate those predictions that also reproduce the state-defining pocket geometry, indicated by the color coding in Figure 4 and shown in detail in Figure 1B,E,H. Recovery of the state-defining geometry was therefore assessed by visual inspection of each condition’s representative model superimposed onto the reference structure, comparing the different conformations of the state-defining residues. Interaction fingerprints, computed independently for every condition, quantify how much of the reference contact network is reproduced (Figures S5–S11). LRRK2 is the most informative case, since its upward Y2018 orientation is not represented in earlier structures: all conditions that reproduced the global fold reach RMSDs below 1.3 Å, but the default predictions retained the canonical tyrosine arrangement, whereas both state-biased AlphaFold3 conditions recovered the noncanonical orientation together with the corresponding DYG backbone conformation, irrespective of whether the bias was introduced through the DFG-out MSA or through the DFG-out template (Figure 4). Chai-1 resulted in an upward direction of the tyrosine side chain in all conditions where the MSA was removed, but flipped the backbone conformation so the DYG motif as a whole was not recovered. None of the four templates supplied for LRRK2 (Table S2) shows the upward Y2018 position, so the recovery under the template-only condition cannot be a direct transfer from a template.

However, Boltz-2 with base settings yielded the lowest ligand RMSD for ALK2-ATP, which is the only example in our set that has a closely related ligand present in the training data (ALK2 with AMPPNP in PDB 6UNQ). Although not showing the lowest ligand RMSD, AlphaFold3, and RoseTTAFold3 recovered the correct 1-ethyl orientation in ALK2 bound to RK783 under base settings, with only modest improvement in RMSD upon addition of biased input information (Figure 4).

For the GPCR predictions, backbone accuracy and ligand placement behaved largely independent. Protein RMSD for the inactive-state 5-HT2A receptor was consistently low, including for models in which the ligand was clearly misplaced (Chai-1 predictions), and neither templates nor MSA manipulation substantially altered these results (Figure 5), as the unmodified predictions were already close to the reference fold. Prediction quality decreased overall for the active-state adenosine A_2A_ receptor, where both interventions had limited impact (Figure 5). The poorest protein folds were obtained for the G protein-biased active state of the DOR. Notably, this was the only system in which the interventions changed the outcome: AlphaFold3 with both manipulated MSA and template input produced the best model of the set (Figure 5), suggesting that templating may become relevant primarily where the unassisted prediction fails.

In summary, state-biased inputs helped where the target conformation is absent from the structural record, as for LRRK2 and DOR, and made little difference where it is close to frequent conformations or already present in the training data. For most systems, the difference in ligand RMSD between the best biased and the best default condition was smaller than the spread among diffusion samples within a single condition, and we therefore do not interpret it as a systematic advantage. Improved local geometry also did not extend to the global conformation, as the biased models retained an inactive-like ICL3 in both active-state GPCRs.

### Model Confidence Metrics Partially Report RMSD but Not State Fidelity

When identifying a metric to guide selection of the optimal ligand conformation in the absence of ground-truth structural information, we observed that protein pLDDT showed no clear association with pose accuracy (Pearson r = −0.04, Figure 6 A), whereas ligand pLDDT correlated moderately (r = −0.67, Figure 6 B), indicating that only ligand confidence is indicative of ligand placement. For the protein fold a similar trend is seen where protein pLDDT correlates moderately with the accuracy of protein fold (r = −0.49 Figure 6 C), while ligand pLDDT reflects backbone quality only weakly (r = −0.29 Figure 6 D). Similar analysis for other known reliability metrics (interface pTM, protein–ligand interface PAE, and overall PAE) shows that none of them correlate with protein fold or ligand placement to a meaningful degree (Figure S4).

**Figure 6:**
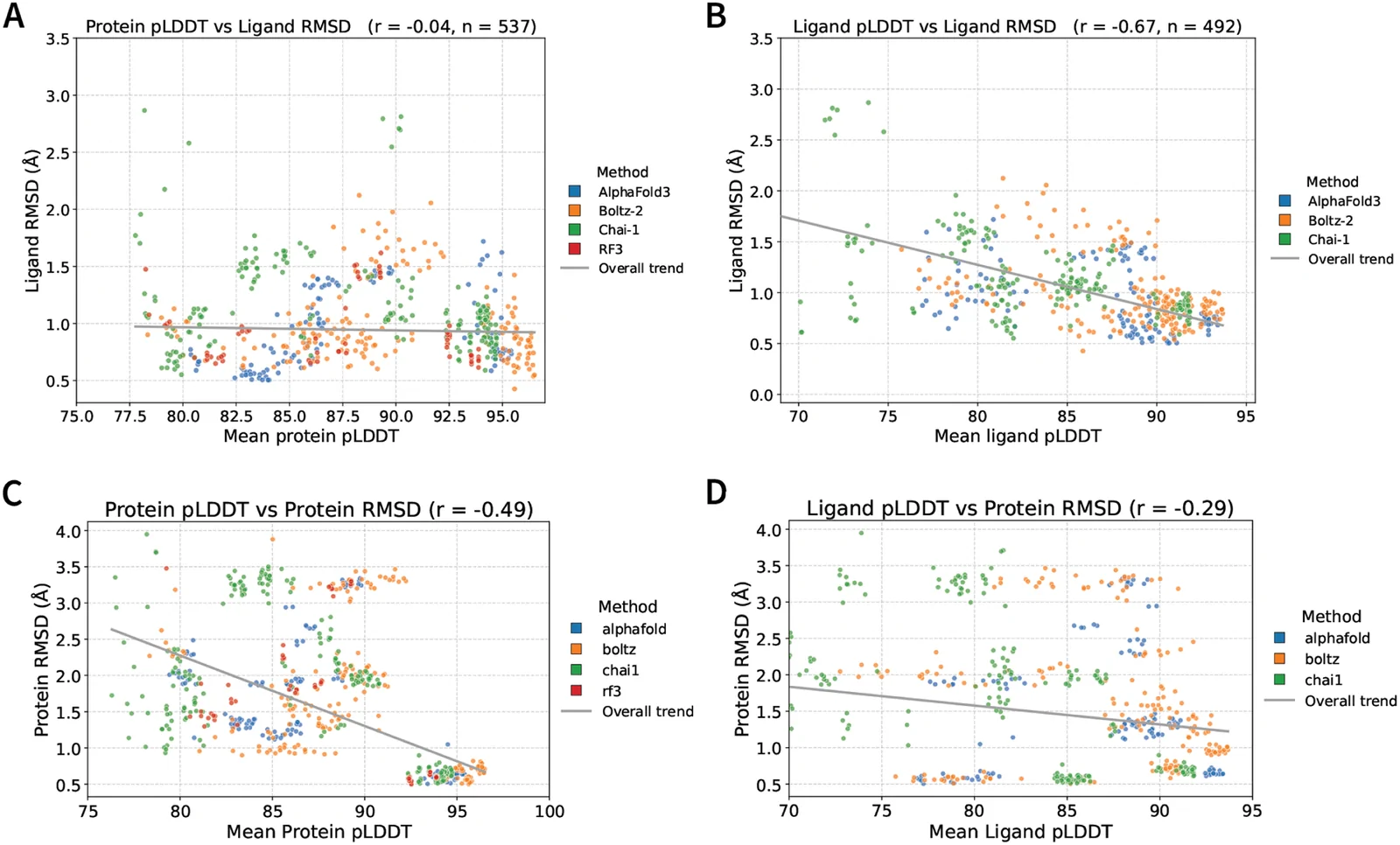
Model confidence (pLDDT) plotted against structural accuracy (RMSD), restricted to near-native predictions so that the reported correlations do not merely separate failed from successful models. **A** Protein pLDDT against ligand RMSD (*n* = 537). **B** Ligand pLDDT against ligand RMSD (*n* = 492). **C** Protein pLDDT against protein RMSD (*n* = 636). **D** Ligand pLDDT against protein RMSD (*n* = 575). RoseTTAFold3 writes a placeholder ligand pLDDT and is therefore excluded from B and D, which accounts for the smaller *n* in those panels.

## Discussion

In this study, we evaluated state-of-the-art protein–ligand cofolding models on systems whose biological function depends on defined conformational states. Because these methods are primarily intended for applications with limited prior structural knowledge, we adopted a general, database-driven biasing strategy based on state annotations from GPCRdb (https://gpcrdb.org/)^6^ and KLIFS (https://klifs.net/)^7^ rather than target-specific manual curation. This design allowed us to probe how current cofolding frameworks behave under conditions that approximate realistic prospective use. Across the evaluated targets, accurate recovery of ligand-bound structures was achievable, but depended strongly on the interplay between model architecture and input biasing. Within the examples tested, state-aware biasing enabled near-native predictions where the local pocket conformation was absent from the training data, whereas comparable accuracy was reached without explicit biasing wherever that conformation was already represented. Additional stochastic sampling via multiple seeds did not improve ligand RMSD or model confidence (Figure S3) although it was suggested by the literature to improve pLDDT of antibody–antigen binding in AlphaFold3.^12^

Because baseline templating failed to improve most predictions, template signals may need to be weighted more strongly to impact cofolding performance. This adjustment could be beneficial when ligands interact with well-established protein conformations. However, for conformationally novel or nonclassical binding sites, excessive template weighting may bias models toward known inactive states, as seen in Boltz-2 with kinases.

The strong misfolding when omitting MSAs in RoseTTAFold3 and AlphaFold3 implies that MSA modifications influence them more strongly and would need to be applied with particular care to avoid destabilizing their predictions. At the same time, any custom MSA editing likely benefits from a more cautious approach if the goal is to preserve and possibly clarify evolutionary signal quality, which resonates with recent work on MSA denoising.^32^ This observation is consistent with recent studies indicating that cofolding accuracy is dominated by strong structural priors, while shallow perturbations of evolutionary input alone often fail to reliably steer predictions.^20^

The kinase examples illustrate that state recovery remains a primary bottleneck uncoupled from correct ligand placement. Binding of RN277 in LRRK2 or ATP in ALK2 both require a defined orientation of a tyrosine, which was only resolved in a subset of predictions suggesting that cofolding models still depend strongly on training-set coverage and are influenced by the frequency of rare conformations within the templates too. For the one novel pocket conformation in our set (LRRK2), ligand placement was accurate but the geometry was recovered only with biased inputs. The limitation of relying solely on filtered template databases becomes evident here: these data sets are enriched for canonical conformations (DFG-in/out), and none of the supplied entries contains the novel orientation or the surrounding backbone arrangement of the reference. Yet the same templates had opposite effects across models. AlphaFold3 recovered the orientation under the template-only condition, whereas Boltz-2 retained the canonical tyrosine arrangement despite accurate ligand placement. Whether a state-annotated template shifts a prediction therefore appears to depend less on its specific content than on how strongly a given framework weights template information against its learned priors, thus possibly also how the templates are weighed by the user during prediction. A similar decoupling of ligand positioning from local backbone adaptation was reported by Masters et al.,^21^ who found that even removal of key binding residues did not substantially alter ligand positioning. This also supports the emerging view that generative structure predictors extrapolate only minimally beyond known conformational space rather than fully sampling it. In line with this, AlphaFold3 enzyme domain motions have been shown to depend strongly on apo/holo representation in the PDB, with ligands exerting only moderate influence on the predicted conformation.^24^

Interestingly, for GPCRs we found that even when the ligand was placed reasonably, the active-state conformation was still consistently pulled toward the inactive state. That mismatch suggests that ligands exert only a modest influence on the global fold prediction and primarily shape the local environment of the binding pocket. A related pattern has been reported for ligand-induced domain motion in enzymes.^24^ This is helpful for interpreting binding interactions but limits the ability of specific ligands to steer the overall conformation in systems like GPCRs. However, suboptimal protein fold seems to affect the prediction of ligand conformations in a more drastic manner, as seen by the DOR example.

Active-state GPCRs appear to be particularly challenging for cofolding models, likely because activation involves substantial structural rearrangements that can differ between receptors. Intracellular loops, especially ICL3, are often unresolved in PDB structures due to their inherent flexibility. This lack of structural data may aditionally make accurate modeling more difficult. For instance, the 5-HT_2A_ receptor has an unusually long ICL3, and the absence of comparable templates for such extended loops could complicate full-length predictions even when the orthosteric ligand is correctly placed. The DOR was modeled with a G protein-biased agonist, ADL5859, which is a type of ligand underrepresented in available cocrystallized structures. As a result, the intracellular conformation induced by this ligand may be uncommon and poorly sampled in template databases. Overall, variability in activation-related rearrangements, unresolved or unusually long intracellular loops, and limited structural coverage of biased ligand-bound states may contribute to the greater difficulty of modeling active-state GPCRs while ligand placement in the orthosteric site remains comparatively robust.

Interestingly, pocket geometry in the GPCR set is recovered with only minimal deviations, in contrast to the kinase systems. This difference may be attributed to the structural anatomy of the two binding sites. The orthosteric GPCR pocket is defined almost entirely by side chains projecting from the rigid extracellular halves of the transmembrane helices, with ECL2 as the only substantial loop contribution, whereas the kinase ATP site is lined to a large extent by flexible loop regions. Notably, the state-defining elements of the GPCR fold are located remote from the ligand, so a well-modeled pocket carries little information about the intracellular state. In kinases, in contrast, the elements that define the conformational state also shape the pocket itself.

In our evaluation, per-residue pLDDT profiles were generally highest across the receptor core and decreased in flexible peripheral regions which we omitted in further analyses (Figure S2). Within the core, ICL3 emerges as one of the most challenging regions to model accurately for GPCRs. This difficulty may stem from the models being trained on a mixture of active and inactive structures, creating ambiguity in this flexible intracellular loop, which undergoes the largest rearrangement during GPCR activation. Attempts to guide predictions through MSA editing or templating proved to be insufficient, especially for the active-state conformation, as there seems to be a preference for ICL3 conformations resembling the inactive state. In general, models tend to predict the inactive-like ICL3 conformation almost perfectly, while their overall protein predictions for active or biased receptors reflects inactive-like or intermediate ICL3 structures. An exception in our evaluations was the DOR, where AlphaFold3 combined with a template and biased MSA correctly predicted ICL3. This suggests that, with additional weighting of these model biases, current methods may be able to better capture relevant protein conformations, ultimately improving the overall accuracy.

In this study, custom templates were selected automatically based on the MSAs generated from the state annotated databases, and no additional criteria were applied beyond this sequence and state information. This means that aspects such as ligand identity or functional context were not explicitly considered during template selection. In principle, the process could be made more deliberate by incorporating searches for templates with similar ligands or, in cases involving biased GPCR agonists, by prioritizing structures that contain ligands with comparable signaling profiles. However, in the case of biased agonists, it remains unclear whether ligand bias is a broadly transferable feature, either across different GPCRs or even among ligands acting on the same receptor. Because of that uncertainty, any attempt to incorporate bias driven criteria into template selection would need to be approached cautiously. Still, it points to a possible way of broadening template choice beyond the purely sequence based strategy used here.

Furthermore, conformational states that are rare, mutation-dependent, or only accessible through dynamic reorganization are not recovered without explicit conditioning. In our study this was the case for the allosteric ligand regulated by cryptic pocket opening^26^ and less conventional activation types such as biased agonists. Since they are markedly underrepresented in the structural data that most cofolding models were trained on, the models may not have a strong prior for how an allosteric pocket should be occupied or how its engagement reshapes the surrounding conformation. This limitation is consistent with recent analyses showing that cofolding models tend to overfit to well-represented protein–ligand interactions and struggle to generalize to uncommon binding sites or underrepresented conformational states, including allosteric pockets.^20^ In situations like this, using templates that contain a ligand bound in the relevant allosteric site could, at least in principle, help guide the model toward a more accurate arrangement of that region. This does not alter the overall workflow, but it highlights how template selection could be adjusted when the available structural landscape is sparse.

Cross-model comparisons under curated, state-specific MSAs used shared alignments across all frameworks and therefore primarily compare model behavior. In this comparison, the default MSA condition is not directly harmonized, because only AlphaFold3 used its internal MSA pipeline; performance differences under this setting may therefore partly reflect MSA generation rather than architecture alone. The analysis reported here should therefore be read as the behavior of each framework under its respective default or recommended MSA-generation route, which we consider the most realistic setting for prospective use.

The generality of the observed decoupling between ligand placement accuracy and protein conformational accuracy is bounded by the scope of our benchmark. Our evaluation rests on seven representative systems from two protein families, and we therefore present this decoupling as a consistent trend within the evaluated scope. Kinases and Class A GPCRs were selected because both offer unambiguous, structurally well-defined conformational state definitions (DFG-in/out and αC-in/out; inactive, intermediate, and active), which is a prerequisite for assessing state-specific accuracy, and because both are densely represented in the PDB and, by extension, in the training data of the evaluated models. This makes these systems comparatively clear test cases, and we would expect the decoupling to be at least as pronounced on targets with sparser structural coverage, although this remains to be demonstrated. Whether the decoupling extends to fold classes with less discretely defined conformational states or different functional plasticities, such as transporters or multidomain systems undergoing large-scale motions, has yet to be quantitatively assessed and would require a systematic evaluation across these target spaces.

In conclusion, cofolding approaches appear promising due to their ease of use and adaptive fitting of the binding pocket to the ligand, which is lacking in many docking approaches. However, the overall reliability of the resulting protein conformations remains questionable, particularly with respect to state-specific accuracy. Incorporation of state-relevant templates and multiple sequence alignments improved local geometry in individual cases, but did not enforce the correct conformational state. Careful ligand selection is crucial, as cofolding performs rather robustly for orthosteric ligands in our case studies, while it consistently misplaced the one allosteric binder tested, highlighting a current limitation that must be considered when interpreting the results.

## Methods

### State-Specific MSA and Template Generation

For GPCRs, active and inactive state structures were retrieved from the PDB as mmCIF files, based on their state annotations in the GPCRdb (https://gpcrdb.org/).^6^ For kinases, the entire KLIFS database (https://klifs.net/)^7^ was downloaded, representing the most comprehensive collection of human kinases. PDB entries were sorted according to their conformational states, including DFG-out, DFG-inter, Active, C-Helix-out, and Salt-Bridge-out, using Kincore.^33^ The selected PDB files were then converted to mmCIF format. Using Biopython,^34^ FASTA sequences corresponding to the preferred chain for each entry were generated. MSAs of each subdatabase containing one conformational state were subsequently produced using MMseqs2 ^35^ saved as.a3m files using the result2 msa command line option.

Custom templates were chosen by using the mmCIF structure files of the top four hits in each individual state-specific MSAs. The structures supplied as custom templates and the depth of the state-specific alignments used for the biased conditions are listed in Table S2 in the supplements.

### Unbiased MSAs and Template Generation

For Boltz-2 and Chai-1, MSAs were obtained via their MSA-server mode, which submits query sequences to the public ColabFold MMseqs2 server (https://api.colabfold.com/).^12,14,36^ As RoseTTAFold3 does not provide a built in MSA generation command, the MSAs were generated separately with the standalone ColabFold Docker image in msa-only mode using default settings. AlphaFold3 generated MSAs with its internal data pipeline, using Jackhmmer and Nhmmer searches against the local AlphaFold3 sequence databases^12^ downloaded 17.05.2025. Among these methods, only Chai-1 offers a template-server mode. When enabled, template hits are retrieved from the ColabFold MMseqs2 server in the form of a all_chain_templates.m8 file that is parsed by the Chai-1 template loader.

### Input and Biasing Data

Training-data cutoffs differ between the four models: AlphaFold3 and Chai-1 apply release-date cutoffs of 30.09.2021 and 12.01.2021,^12,15^ Boltz-2 of 01.06.2023,^14^ and RF3 of 01.09.2021.^16^ The six inhibitor- and agonist-bound reference structures were released between November 2023 and June 2025 (8TSD, 22.11.2023; 8ZMG, 28.08.2024; 8Y45, 27.11.2024; 8UGW, 18.12.2024; 9DMI, 18.06.2025; 9L04, 25.06.2025) and therefore postdate the release-date cutoffs. The AMPPNP-bound ALK2 structure (6UNQ) was included as one example for a complex most models are expected to have seen: released 07.07.2021, which lies within the training window of AlphaFold3, Boltz-2, and RF3. An overview table can be found in the supplements as Table S1.

### Inference

For each method and condition, the general inference settings were kept similar using one seed (″0″)/no seed for Boltz-2 and five diffusion samples. For AlphaFold3 predictions were repeated with five seeds to evaluate the stochastic effects during sampling (Figure S3). Further inference settings were kept on default if not indicated.

#### AlphaFold3

Five setups were used in AlphaFold3 for each protein. Custom MSAs were first processed as AlphaFold3 requires a specific formatting. Therefore, the state-specific MMseqs2 A3M alignments were normalized with reformat.pl (HH-suite v3.3.0 ^37^) using the -M first option, which filters sequences so that all entries contain the same number of nonlowercase characters as the query sequence. The processed MSAs were then provided to AlphaFold3 within the JSON files by setting the ″unpairedMsaPath″ entry and providing an empty string to ″pairedMsa″. In the *no MSA* conditions, the ″unpairedMsaPath″ was set to an empty string too, while the *base settings* condition lacks the JSON field entirely, resulting in AlphaFold3 automatically generating MSAs with the internal data pipeline.

In the conditions containing templates, the top 4 MSA hits were used as templates. Therefore, a python script was created that extracts sequences from mmCIF files and aligns the query and template sequences using BioPython^34^ before calculating the queryIndices and templateIndices arrays for the JSON input file. AlphaFold3 does not intend to combine an unbiased MSA with biased templates within one run, which is why we only generated 5 conditions for alphaFold3. To generate five diffusion samples, we used the --num_diffusion_samples = 5 flag. All models were generated with AlphaFold3 v3.0.1.^12^

#### Boltz-2

Boltz-2 ^13,14^ models were generated using six different setups. The *unbiased* models were created using the base input, which included the protein sequence and ligand SMILES, with multiple sequence alignments (MSAs) generated through the --use_msa_server flag. The *no MSA* models were produced by explicitly disabling MSA usage, setting the MSA input to ″empty″. For the *template* condition, templates were selected from the top four hits identified in the MSA of the annotated database. The *biased MSA* models used a manually curated A3M file as input, generated according to the procedure described in the MSA generation section. For each condition, diffusion samples were set to five via the --diffusion_samples 5 flag during inference. All models were generated with Boltz-2 version 2.2.0.^13,14^

#### Chai-1

For Chai-1, state-specific A3M MSAs were converted to Parquet format using chai-lab a3m-to-pqt and the resulting directory was supplied via the –msa-directory option during inference. Templates were provided through M8 alignment files, which define the correspondence between the query sequence and full-length protein data bank entries retrieved by the model. Because the custom templates from KLIFs database consist only of the kinase domains, the subject start and end indices were set to the kinase domain boundaries (0-based). To generate five diffusion samples –num-diffn-samples 5 was added to the command during inference. All models were generated with Chai-1 version 0.6.1

#### RoseTTAFold3

For RoseTTAFold3 biased MSAs, the previously described A3M files were adjusted with a python script that strips lowercase insertions, enforces a fixed postprocessed length equal to the query, and rewrites all sequences in single-line format. The colabfold-MSA were generated with ColabFold. Except for the condition without MSA, the resulting A3M files were supplied to RF3 via absolute msa_path entries in the JSON files. As RoseTTAFold3 defines templating different than the other methods, templates were not considered. To generate five diffusion samples the ––diffusion_batch_size = 5 flag was used. All models were generated with RoseTTAFold3 (commit b5c9685c7) using the model rf_921.pt (training cutoff 09/21) downloaded on 08.10.2025.^16^

### Analysis

The generated cofolding structures were aligned to their corresponding crystal structure (mmCIF) using PyMOL 3.1.0 and protein RMSD was calculated simultaneously on backbone atoms (N, Ca, C, O) and with iterative outlier rejection disabled (cycles = 0) to ensure the reported RMSD covers the stated selections. For GPCRs and for LRRK2, alignment and protein RMSD calculations were restricted to the folded receptor core to exclude highly flexible terminal and loop regions (69–397 for 5-HT2A, 1–314 for A2AR, 39–335 for DOR, and 1879–2138 for LRRK2). Each condition contributes the five diffusion samples of one seed. AlphaFold3 additionally writes its top-ranked model a second time outside the sample directories, and this duplicate was excluded, giving 105 models per target. Subsequently, the aligned complexes were saved as a PDB file. Ligand RMSD was evaluated with MDAnalysis 2.10.0 on the ligand heavy atoms in the protein-aligned reference frame. The representative model of each condition shown in plots is the one with the highest mean protein pLDDT across the five diffusion samples and the ligand RMSD shown derive from that same model using the center = False, superposition = False flags. Per-residue pLDDT profiles were generated using the highest-mean-pLDDT structures. Across conditions, structures shown in three dimensions were selected by visual inspection of the binding-site geometry, considering recovery of the main interactions alongside ligand RMSD. For calculation of confidence metrics varying numbers of structures are used in the figures as not every model reports every metric: RoseTTAFold3 writes a placeholder ligand pLDDT and Chai-1 does not store PAE by default.

## Supplementary Material



## Data Availability

The ground truth structures are available through the RCSB Protein Data Bank (https://www.rcsb.org/)^38^ under the accession codes: for GPCRs: 8ZMG,^29^ 8UGW,^30^ and 8Y45;^31^ and kinases: 6UNQ,^28^ 9L04,^27^ 9DMI^25^ and 8TSD.^26^ The generated cofolding structure models and analysis scripts are available via Zenodo (10.5281/zenodo.22208005).

## Notes

Supporting Information for “Benchmarking Protein–Ligand Cofolding Models: Correct Ligand Placement Is Decoupled from Accurate Protein Conformation”. Per-Residue pLDDT of Cofolding Kinase Predictions (Figure S1). Per-Residue pLDDT of Cofolding GPCR Predictions (Figure S2). Comparison of Predictions with Multiple Different Seeds (Figure S3). Additional Confidence Metrics and Their Influence on Recovery of the True Structure (Figure S4). Ligand Validity and Interaction Analysis (Figures S5–S11). Training and Biasing Data of Selected Cofolding Systems (Tables S1 and S2) (PDF)
