# Supplementary material for: Benchmarking Protein–Ligand Cofolding Models: Correct Ligand Placement Is Decoupled From Accurate Protein Conformation
Source: J Chem Inf Model. 2026 Oct 20;66(21):14188–99. doi: 10.1021/acs.jcim.6c02169 (PMC13753516; doi:10.1021/acs.jcim.6c02169)
Supplement: Supplementary file 1 [file ci6c02169_si_001.pdf]

Supporting Information of "Benchmarking Protein–Ligand Cofolding Models: Correct Ligand Placement Is Decoupled from Accurate Protein Conformation"

Leon Obendorf,[†,‡] Niklas Piet Doering,[†,¶] Petra Knaus,[*,‡] and Gerhard Wolber[*,¶]

†*equally contributed to this work*

‡*Department of Biology, Chemistry and Pharmacy, Institute of Biochemistry, Signal Transduction Group, Freie Universität Berlin, Thielallee 64, 14195 Berlin, Germany*

¶*Department of Biology, Chemistry and Pharmacy, Institute of Pharmacy, Molecular Design Group, Freie Universität Berlin, Königin-Luise-Str. 2+4, 14195 Berlin, Germany*

E-mail: knaus@zedat.fu-berlin.de; gerhard.wolber@fu-berlin.de

Phone: +49 30 838 52935; +49 30 838 52686. Fax: +49 30 838 452936; +49 30 838 452686

Contents

1 Per-Residue pLDDT of Cofolding Kinase Predictions S3

2 Per-Residue pLDDT of Cofolding GPCR Predictions S4

3 Comparison of Predictions with Multiple Different Seeds S5

4 Additional Confidence Metrics and Their Influence on Recovery of the True Structure S6

5 Ligand Validity and Interaction Analysis S7

6 Training and biasing data of selected cofolding systems S15

1 Per-Residue pLDDT of Cofolding Kinase Predictions

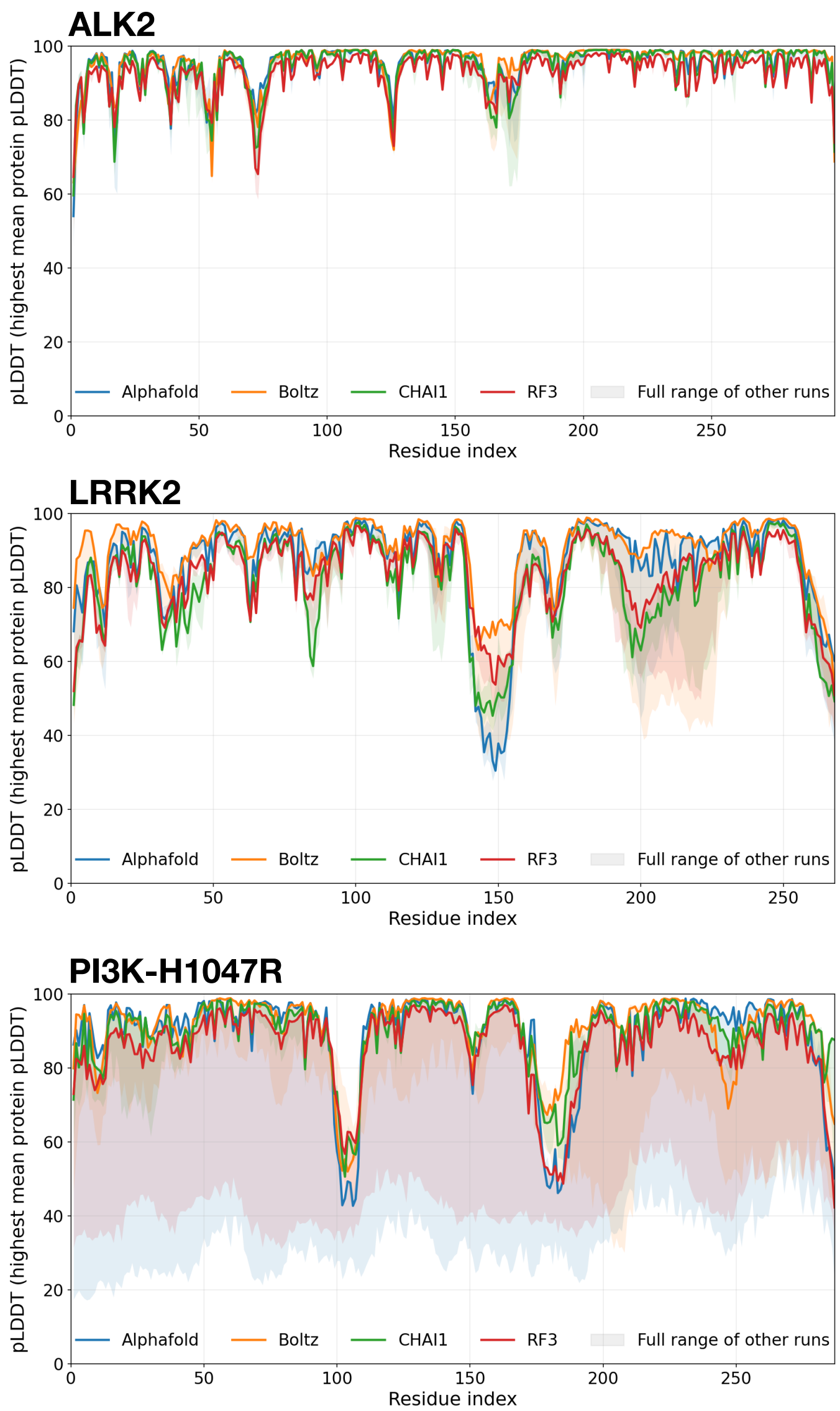

Figure S1: Per-residue pLDDT line-profiles for the best single kinase structure per method (colored lines), selected by highest mean protein pLDDT. The color-shaded region shows the full pLDDT range (min–max) of the highest mean pLDDT structure values across all other cofolding conditions (besides the failed AlphaFold and RoseTTAFold3 no MSA / no Templates) for each residue position. Residue indices are shown on the x-axis; pLDDT scores (0–100) are on the y-axis.

2 Per-Residue pLDDT of Cofolding GPCR Predictions

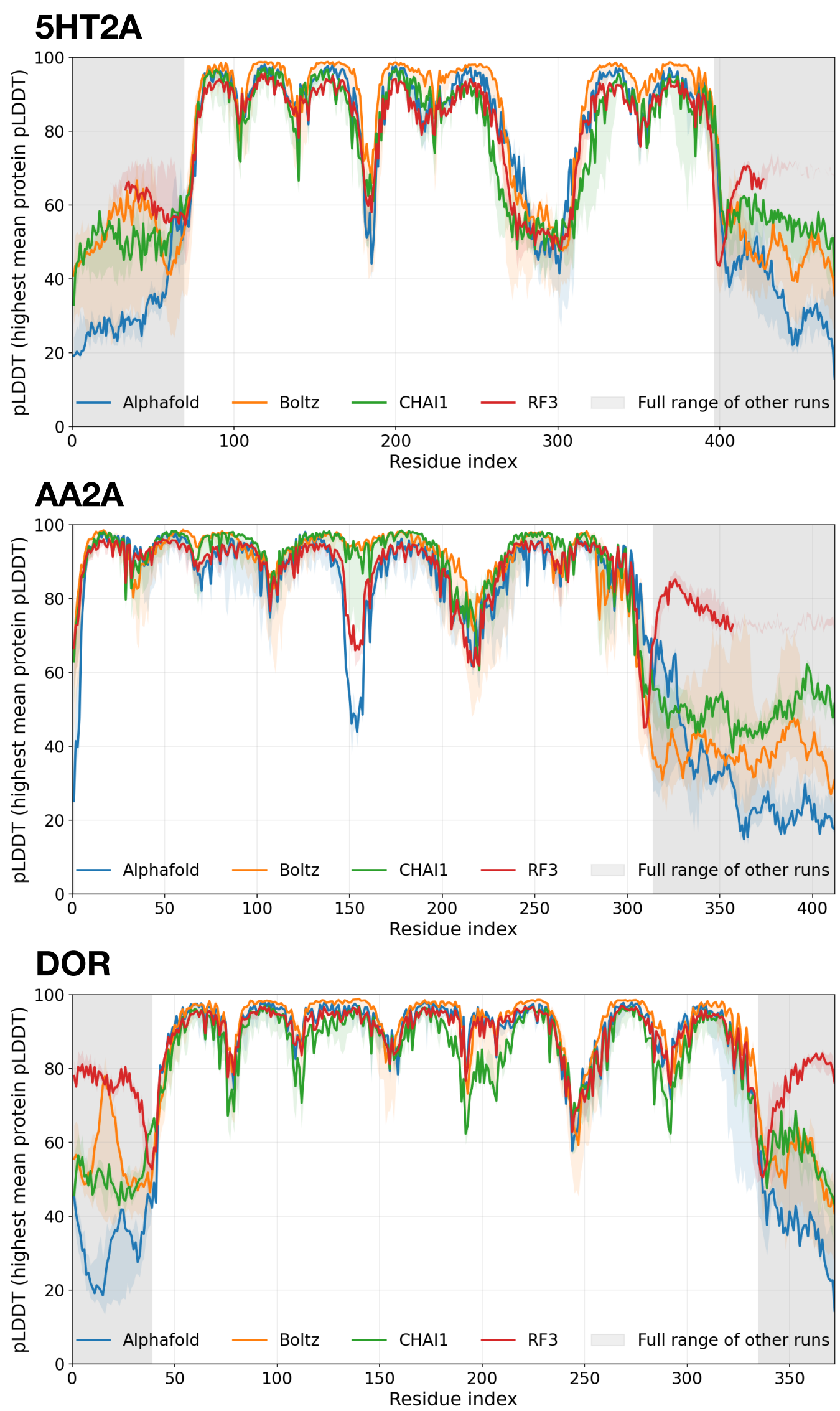

Figure S2: Per-residue pLDDT line-profiles for the best single GPCR structure per method (colored lines), selected by highest mean protein pLDDT. The color-shaded region shows the full range (min–max) of pLDDT values across highest-mean pLDDT structures of all co-folding conditions (besides the failed AlphaFold and RoseTTAFold3 no MSA / no Templates) for each residue position. Residue indices are shown on the x-axis; pLDDT scores (0–100) are on the y-axis. The grey shaded region shows flexible parts of the GPCRs which were not used during alignment and comparison of plDDT values, to only compare the core GPCR besides N- or C-terminal loops.

3 Comparison of Predictions with Multiple Different Seeds

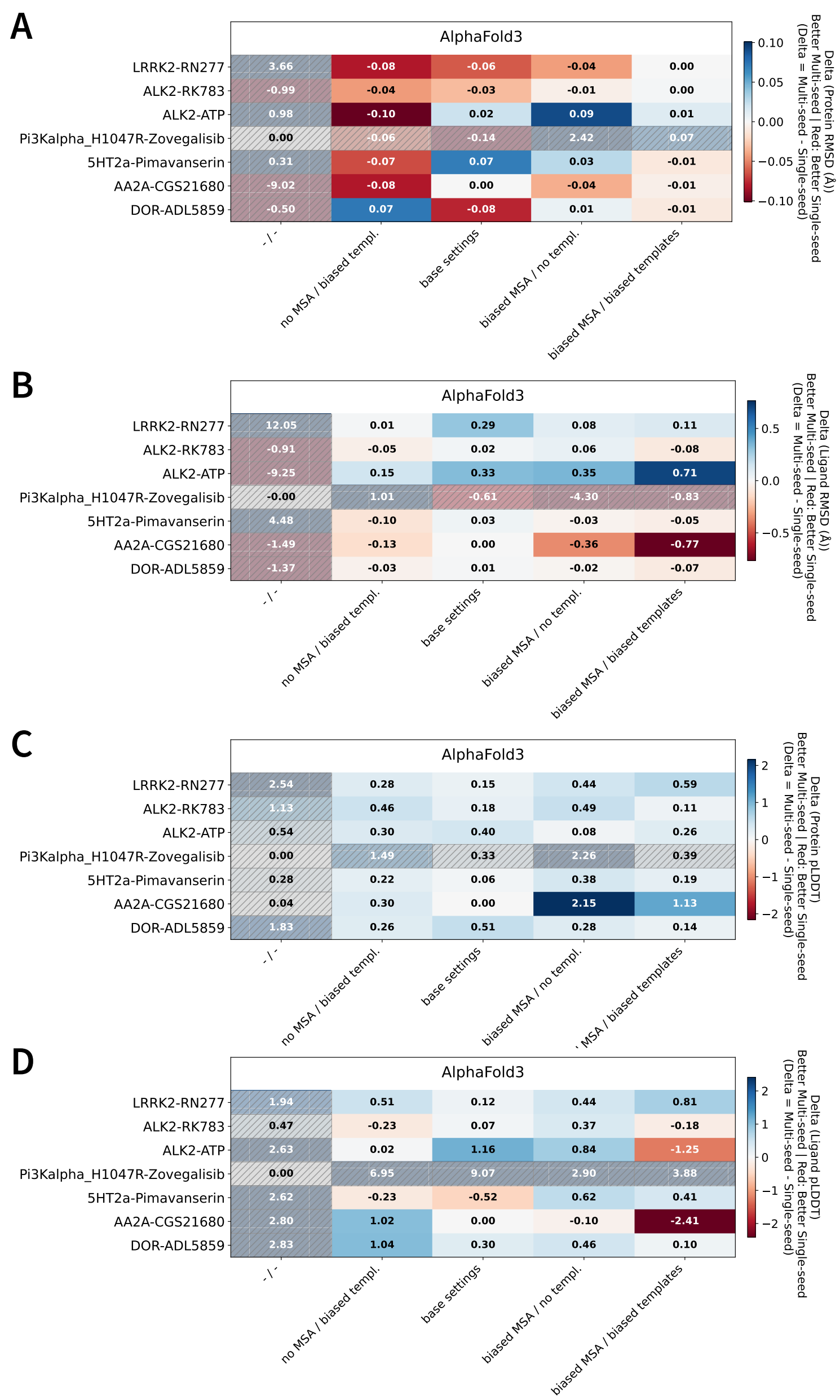

Figure S3: Sensitivity of co-folding outcomes to number of seeds used. Comparison of single-seed and multi-seed predictions for all analyzed targets and prediction settings, grouped by method. **A** Protein backbone RMSD, **B** ligand RMSD, **C** mean protein pLDDT, and **D** mean ligand pLDDT.

4 Additional Confidence Metrics and Their Influence on Recovery of the True Structure

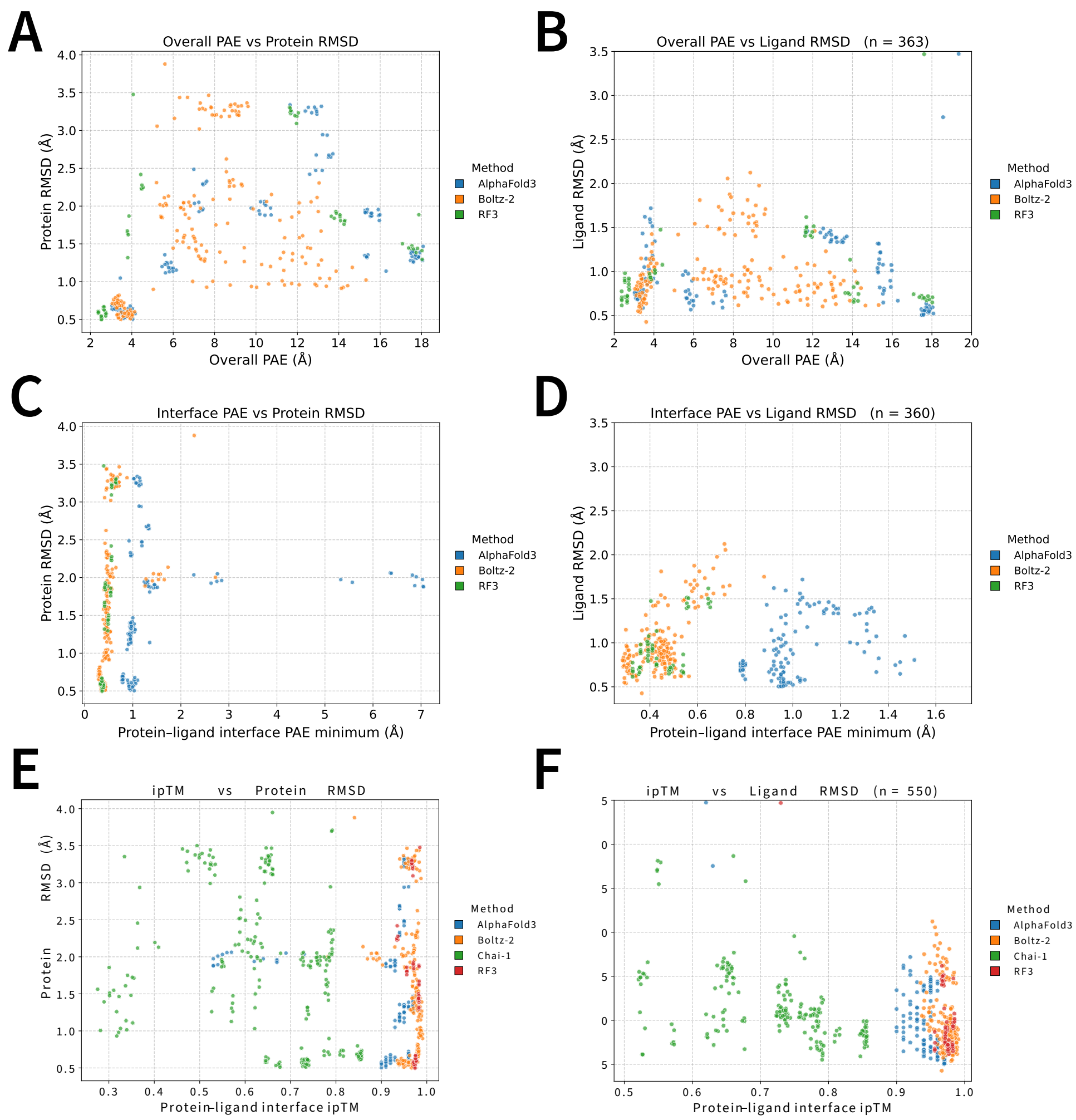

Figure S4: Comparison of the interface-reliability measures with ligand and protein RMSD over the same models: overall PAE (A, B), protein–ligand interface PAE (C, D) and ipTM (E, F), each plotted against protein RMSD (A, C, E; cropped at 4 Å) and against ligand RMSD (B, D, F). IpTM retains possibly a weak association with pose accuracy among near-native predictions (F), interface PAE too (D) and overall PAE none (B). For all dispersion is high. PAE is available for 490 of the 735 models, as Chai-1 does not output it (E, F therefore include Chai-1, A–D do not)

5 Ligand Validity and Interaction Analysis

Interaction fingerprints were computed for all seven co-folding targets, comparing each experimental complex against the best predicted sample of every setting. Ligand bond orders and formal charges were assigned from the corresponding SMILES using RDKit,[1] with the template first corrected to its dominant protonation state at pH 7.4 using Open Babel.[2] Protein heavy atoms and side chains were completed with PDBFixer,[3] without bridging unresolved crystallographic gaps, and bond connectivity was taken from OpenMM[3] residue templates rather than distance-based perception. Interactions were detected with ProLIF 2.2.1.[4] Hydrogen bonds were detected with ProLIF's `ImplicitHBDonor` and `ImplicitHBAcceptor` terms, which infer hydrogen bonding from heavy-atom geometry rather than from explicit hydrogen positions, to avoid problems caused by non manual structure editing. Pose physical plausibility was assessed independently with PoseBusters 0.6.5[5] in `dock` mode, applying all 22 chemistry, geometry, internal-energy and protein-clash checks to the same bond-order-corrected molecules used for fingerprinting.

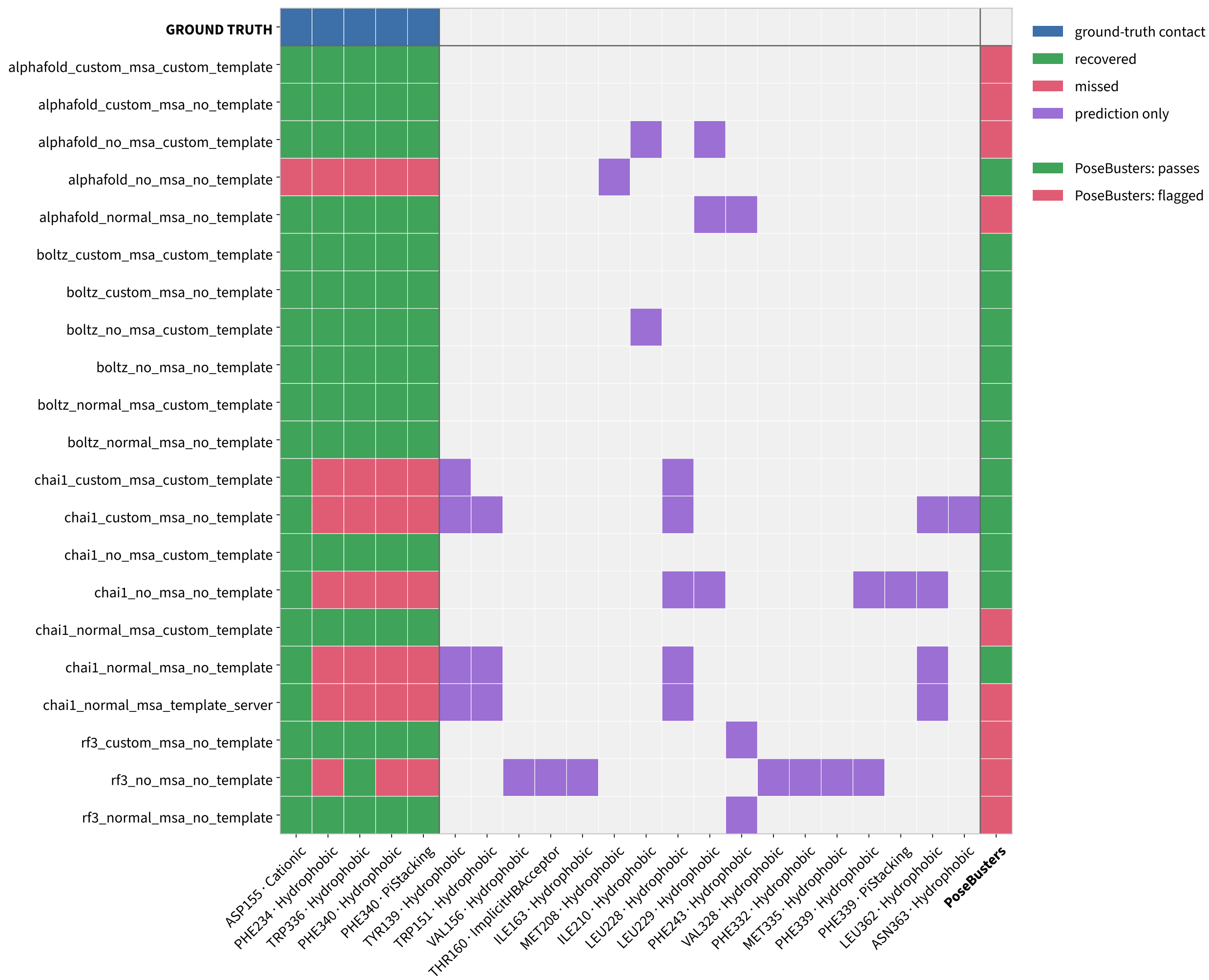

Figure S5: Interaction fingerprints for the 5-HT_{2A} receptor with the antagonist. Rows are the crystal structure (top) and the best predicted sample of each of the 21 settings; columns are individual (residue, interaction type) contacts. The last column reports PoseBusters physical plausibility as "flagged" or "passed" for the ligand in each prediction.

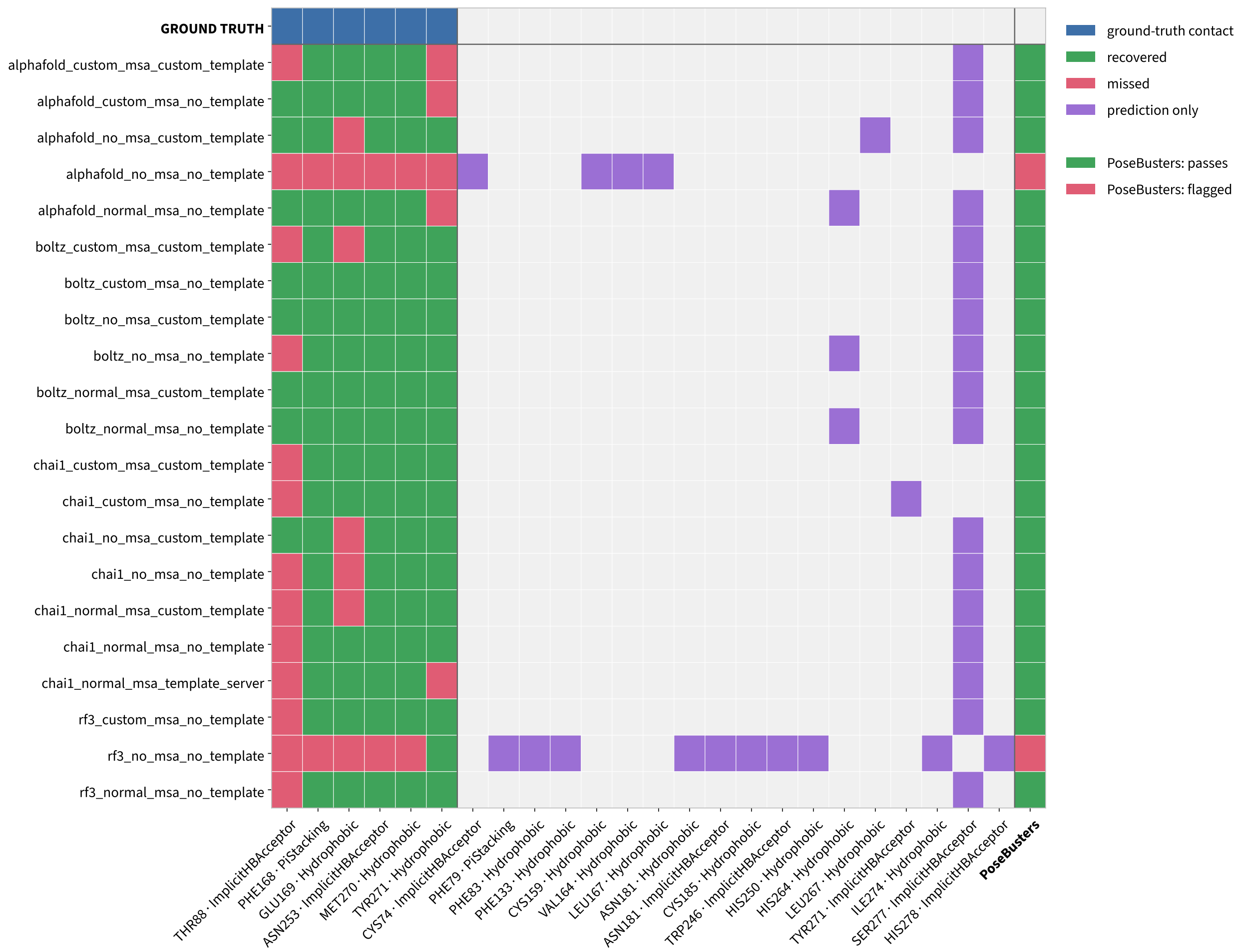

Figure S6: Interaction fingerprints for the adenosine A_{2A} receptor with the agonist. Rows are the crystal structure (top) and the best predicted sample of each of the 21 settings; columns are individual (residue, interaction type) contacts. The last column reports PoseBusters physical plausibility as "flagged" or "passed" for the ligand in each prediction.

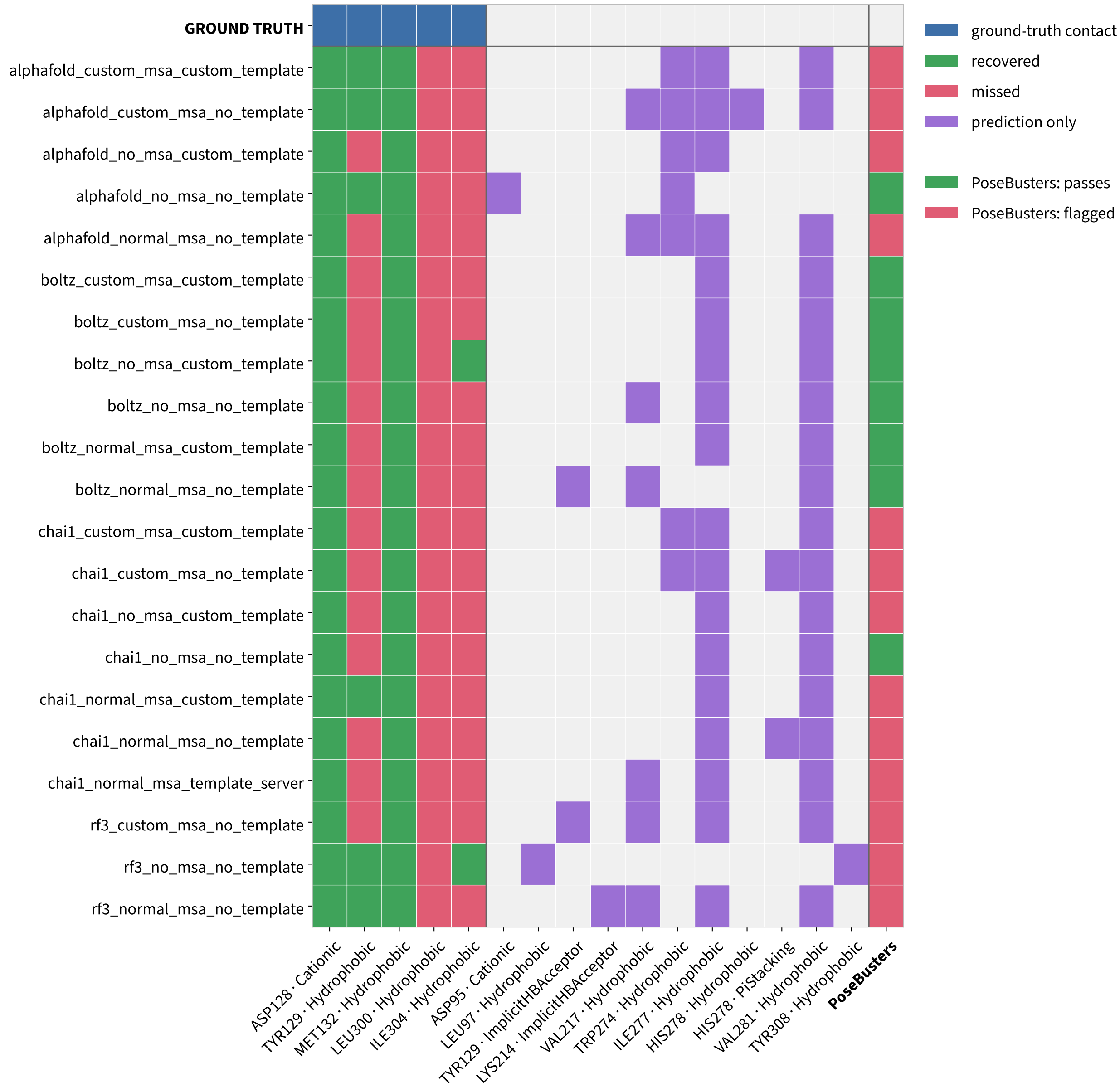

Figure S7: Interaction fingerprints for the δ-opioid receptor with the biased agonist. Rows are the crystal structure (top) and the best predicted sample of each of the 21 settings; columns are individual (residue, interaction type) contacts. The last column reports PoseBusters physical plausibility as "flagged" or "passed" for the ligand in each prediction.

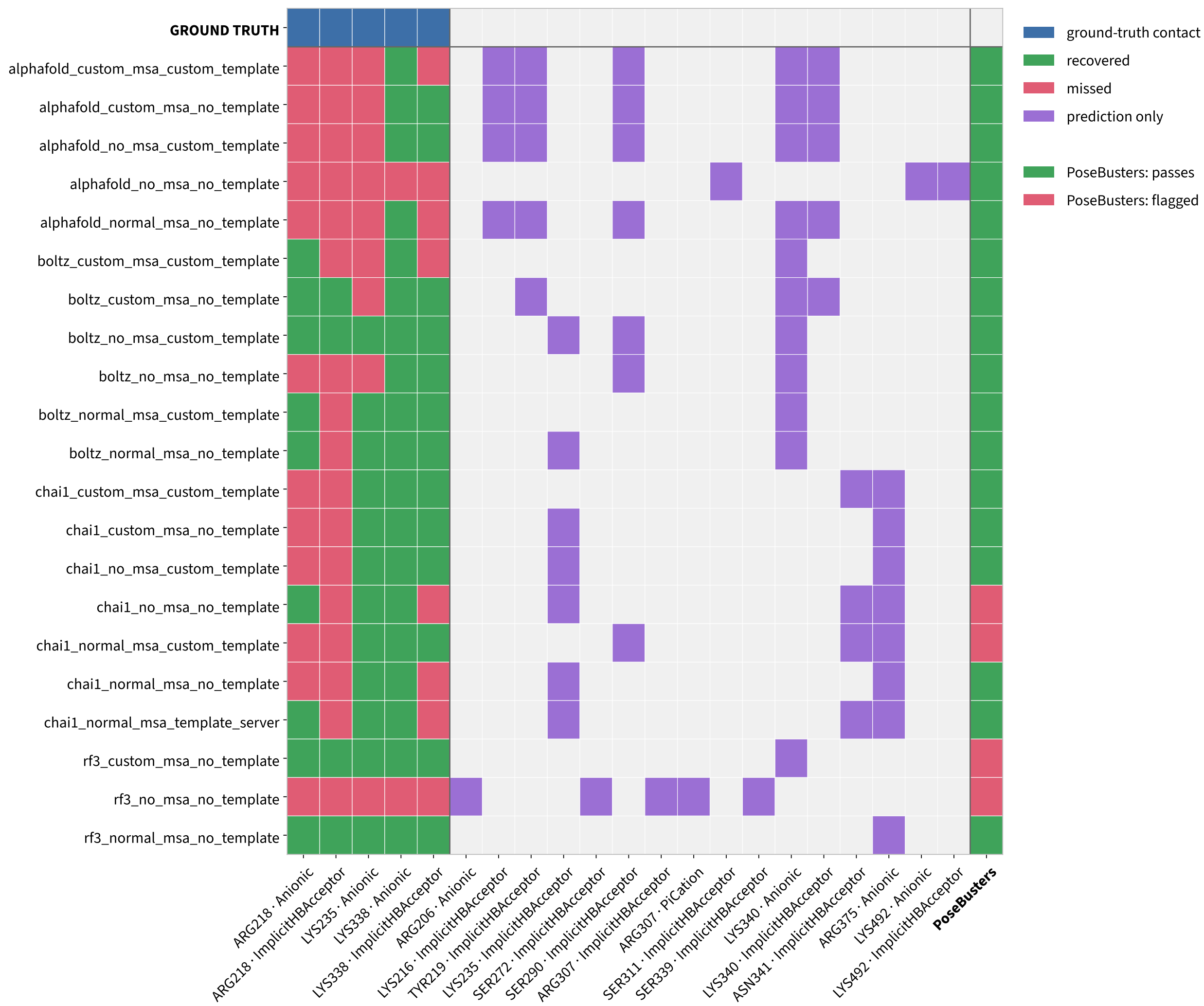

Figure S8: Interaction fingerprints for the ALK2 kinase domain with ATP, compared against the AMP-PNP complex. Rows are the crystal structure (top) and the best predicted sample of each of the 21 settings; columns are individual (residue, interaction type) contacts. The last column reports PoseBusters physical plausibility as "flagged" or "passed" for the ligand in each prediction.

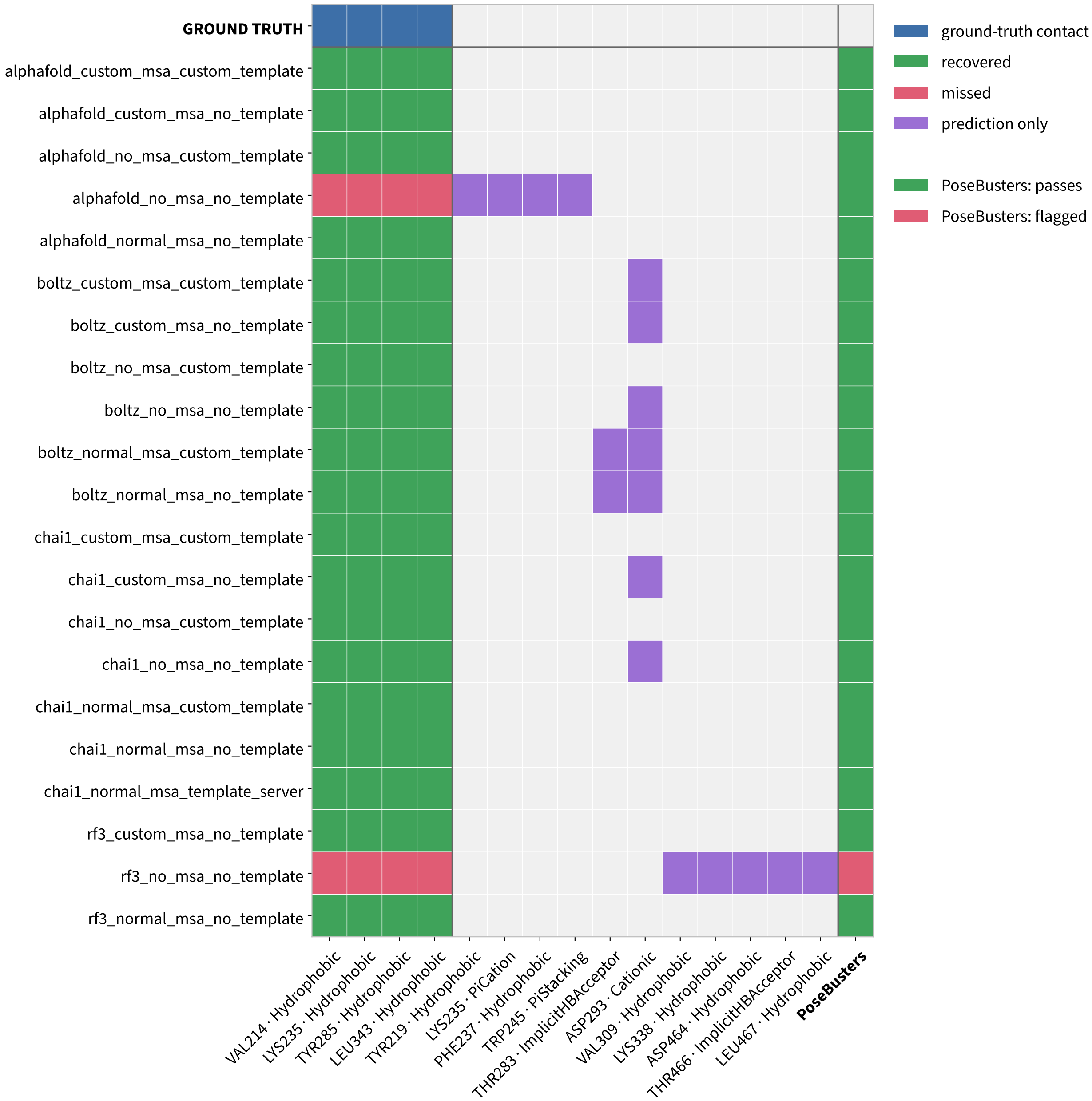

Figure S9: Interaction fingerprints for the ALK2 kinase domain with the type-I inhibitor RK-783. Rows are the crystal structure (top) and the best predicted sample of each of the 21 settings; columns are individual (residue, interaction type) contacts. The last column reports PoseBusters physical plausibility as "flagged" or "passed" for the ligand in each prediction.

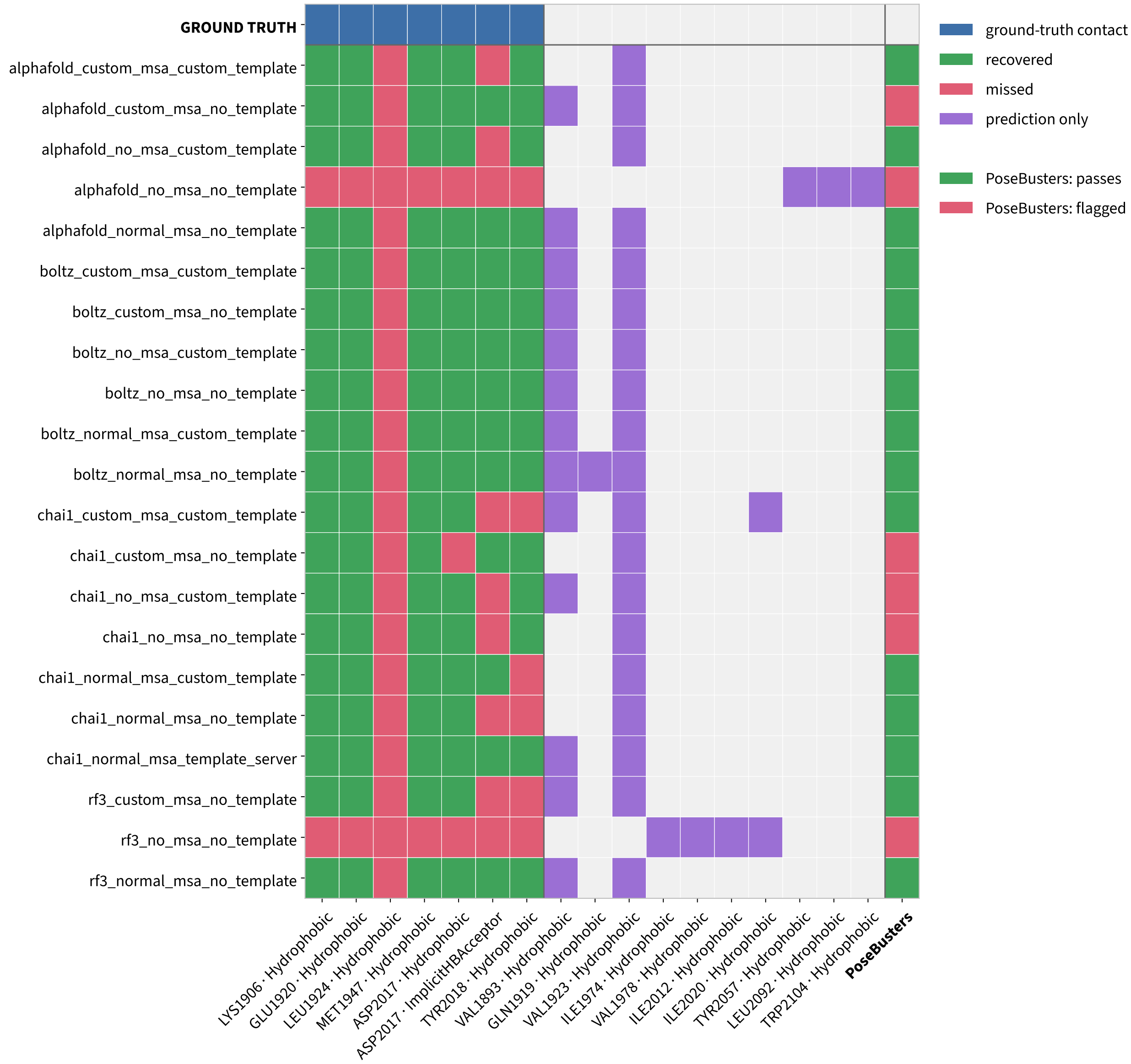

Figure S10: Interaction fingerprints for the LRRK2 kinase domain with the type-II inhibitor RN-277. Rows are the crystal structure (top) and the best predicted sample of each of the 21 settings; columns are individual (residue, interaction type) contacts. The last column reports PoseBusters physical plausibility as ”flagged” or ”passed” for the ligand in each prediction.

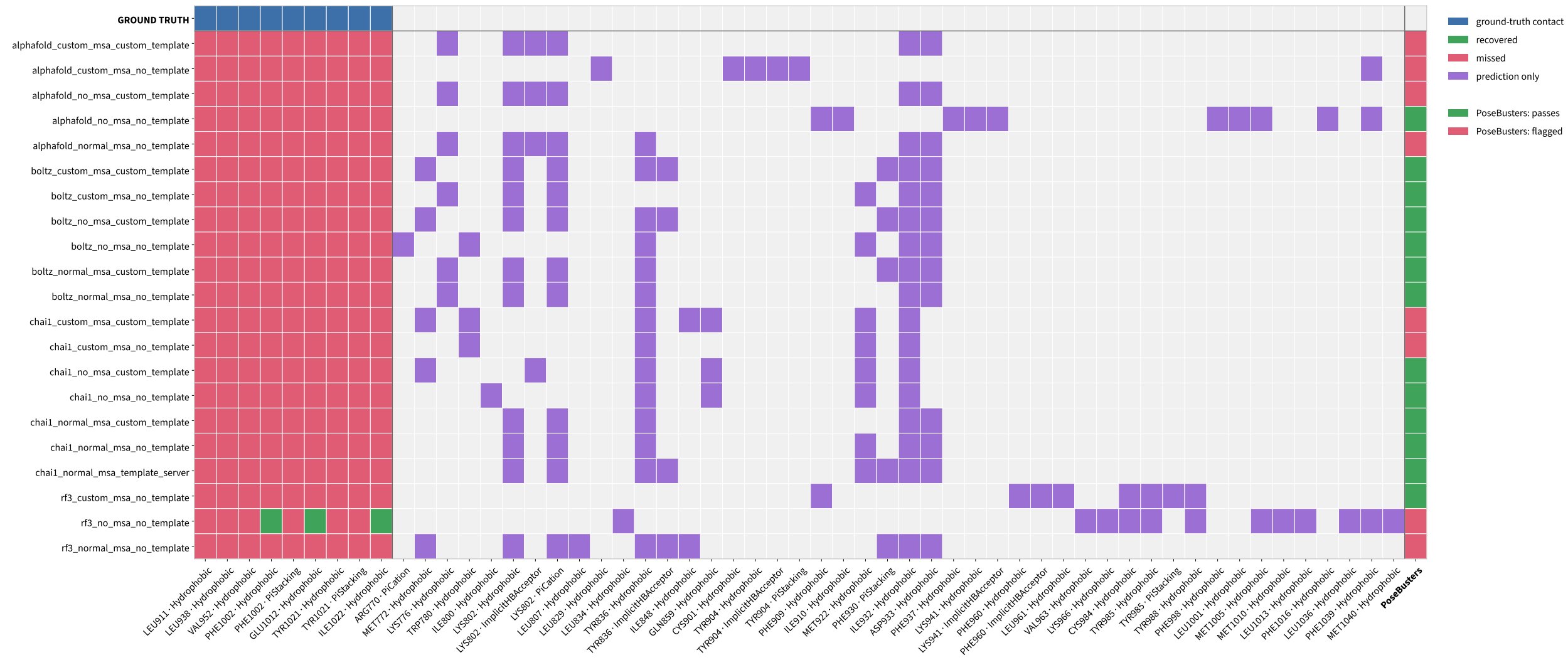

Figure S11: Interaction fingerprints for the PI3K^{H1047R} mutant bound to the allosteric inhibitor Zovegalisib. Rows are the crystal structure (top) and the best predicted sample of each of the 21 settings; columns are individual (residue, interaction type) contacts. The last column reports PoseBusters physical plausibility as "flagged" or "passed" for the ligand in each prediction.

6 Training and biasing data of selected cofolding systems

Table S1: PDB structures used and their inclusion within the training datasets of different structure prediction models. "in" indicates that the target structure is included in the respective training dataset, "out" that it is not. Model columns are labeled with the training-set cutoff date.

			Training-set cutoff			
Target	PDB	Released	AF3 30.09.2021	Chai-1 12.01.2021	Boltz-2 01.06.2023	RF3 01.09.2021
LRRK2	9DMI	18.06.2025	out	out	out	out
ALK2–RK783	9L04	25.06.2025	out	out	out	out
5-HT_{2A}	8ZMG	28.08.2024	out	out	out	out
DOR	8Y45	27.11.2024	out	out	out	out
A_{2A}	8UGW	18.12.2024	out	out	out	out
PI3K	8TSD	22.11.2023	out	out	out	out
ALK2–AMPPNP	6UNQ	07.07.2021	**in**	out	**in**	**in**

Table S2: Structural data used to bias the predictions. State = conformation the bias was built for; custom templates = PDB entries supplied to the model; MSA depth = number of sequences in the state-specific alignment. The same alignments were supplied to AlphaFold3 and RoseTTAFold3. All alignments are deposited with the analysis scripts.

Target	State	Custom templates (PDB)	MSA depth
**GPCRs**			
5-HT$_{2A}$	inactive	6A93, 6A94, 7WC4, 7WC5	208
A$_{2A}$	active	2YDO, 2YDV, 4UHR, 8WDT	301
DOR	active, biased	6PT2, 6PT3, 7T2G, 8F7R	292
**Kinases**			
ALK2–AMPPNP	active	1IAS, 2X7O, 6T6D, 6UNS	1000
ALK2–RK783	active	1IAS, 2X7O, 6T6D, 6UNS	1000
LRRK2	DFG-out	7LHW, 8FO8, 8TZF, 8U7L	669
PI3K	DFG-out	7MYN	2

References

(1) Landrum, G. RDKit: Open-Source Cheminformatics. `https://www.rdkit.org`, (accessed 2024, November 1).

(2) O'Boyle, N. M.; Banck, M.; James, C. A.; Morley, C.; Vandermeersch, T.; Hutchison, G. R. Open Babel: An open chemical toolbox. *J. Cheminf.* **2011**, *3*.

(3) Eastman, P. et al. OpenMM 8: Molecular Dynamics Simulation with Machine Learning Potentials. *J. Phys. Chem. B* **2024**, *128*, 109–116.

(4) Bouysset, C.; Fiorucci, S. ProLIF: a library to encode molecular interactions as fingerprints. *J. Cheminf.* **2021**, *13*.

(5) Buttenschoen, M.; Morris, G. M.; Deane, C. M. PoseBusters: AI-based docking methods fail to generate physically valid poses or generalise to novel sequences. *Chem. Sci.* **2024**, *15*, 3130–3139.
